# Structural alignment of protein descriptors – a combinatorial model

**DOI:** 10.1186/s12859-016-1237-9

**Published:** 2016-09-17

**Authors:** Maciej Antczak, Marta Kasprzak, Piotr Lukasiak, Jacek Blazewicz

**Affiliations:** 1 Institute of Computing Science, Poznan University of Technology, Piotrowo 2, Poznan, 60-965 Poland; 2 Institute of Bioorganic Chemistry, Polish Academy of Sciences, Noskowskiego 12/14, Poznan, 61-704 Poland

**Keywords:** Protein structure, Structural comparison, Combinatorial optimization

## Abstract

**Background:**

Structural alignment of proteins is one of the most challenging problems in molecular biology. The tertiary structure of a protein strictly correlates with its function and computationally predicted structures are nowadays a main premise for understanding the latter. However, computationally derived 3D models often exhibit deviations from the native structure. A way to confirm a model is a comparison with other structures. The structural alignment of a pair of proteins can be defined with the use of a concept of protein descriptors. The protein descriptors are local substructures of protein molecules, which allow us to divide the original problem into a set of subproblems and, consequently, to propose a more efficient algorithmic solution. In the literature, one can find many applications of the descriptors concept that prove its usefulness for insight into protein 3D structures, but the proposed approaches are presented rather from the biological perspective than from the computational or algorithmic point of view. Efficient algorithms for identification and structural comparison of descriptors can become crucial components of methods for structural quality assessment as well as tertiary structure prediction.

**Results:**

In this paper, we propose a new combinatorial model and new polynomial-time algorithms for the structural alignment of descriptors. The model is based on the maximum-size assignment problem, which we define here and prove that it can be solved in polynomial time. We demonstrate suitability of this approach by comparison with an exact backtracking algorithm. Besides a simplification coming from the combinatorial modeling, both on the conceptual and complexity level, we gain with this approach high quality of obtained results, in terms of 3D alignment accuracy and processing efficiency.

**Conclusions:**

All the proposed algorithms were developed and integrated in a computationally efficient tool descs-standalone, which allows the user to identify and structurally compare descriptors of biological molecules, such as proteins and RNAs. Both PDB (Protein Data Bank) and mmCIF (macromolecular Crystallographic Information File) formats are supported. The proposed tool is available as an open source project stored on GitHub (https://github.com/mantczak/descs-standalone).

**Electronic supplementary material:**

The online version of this article (doi:10.1186/s12859-016-1237-9) contains supplementary material, which is available to authorized users.

## Background

Sequencing of genomes of living organisms, that is discovering their linear structure (sequence of nucleotides) is nowadays a fundamental way of acquiring biological data. Such data are then synthesized and analyzed by using computer science tools and methods [1]. One of the consequences of recognizing a DNA sequence of a gene is an attempt to determine the corresponding 3D protein structure. Understanding the process of protein folding is crucial for human health (e.g., drug design, vaccination [2]), because its tertiary structure strictly determines its biological activity in a cell. Over the years, several computational techniques were proposed for predicting secondary [3] and tertiary structures of proteins [4, 5].

Computationally derived protein 3D models exhibit deviations from the corresponding reference structures. Therefore, there is a need to develop structural quality assessment methods that can be used to reliably identify limitations of artificial 3D models in order to choose native-like models, which can be successfully applied in biochemical experiments or in a drug design process. The quality assessment of biological molecules can be performed on the following levels: (1) a global perspective, where for every structural model a single score is computed representing the quality of the whole 3D model, and (2) a local perspective, where a structural reliability score is computed for a local neighborhood of each model residue. In addition, structural quality of a predicted model in comparison with an experimentally derived reference structure can be assessed [6] or computed using a general purpose method for structural comparison of proteins [7]. When the reference structure is not known, the assessment process is much more difficult.

Several methods were proposed to address this challenge in single mode assessment [8, 9] and consensus mode assessment [10–12]. The former ones are based on statistical knowledge derived from known structures and take into consideration mainly physical aspects such as force fields or potentials. The consensus-based methods lead to rank predicted 3D models of a given input protein. They are based on the assumption that the structural region, which is conservative in most of the models predicted by different approaches, can be classified as potentially correct. These methods are often characterized by good performance, but they are hard to use for researchers focused on analysis of a single model. Unfortunately, the performance of single-model-oriented methods is relatively poor in comparison with consensus-based ones [13].

A step toward filling this gap can be an approach dedicated for the single mode assessment problem, which is based on the paradigm of local protein substructures, called *descriptors*. The concept of local descriptors of protein structures was already defined in biological background [14, 15] and applied in several studies demonstrating its usefulness for prediction of residue-residue contacts [16], analysis of structure-function relationships [17] and solving the protein structure alignment problem [18]. The problem considered here is identified as structural comparison of descriptors, which can be used as a crucial component of a knowledge-based potential trained on a wide set of known protein 3D structures. This problem in the scope addressed in this paper was not previously solved in the literature.

Our aim is to provide a new approach and a flexible tool for identification and structural comparison of descriptors toward their application in protein structure assessment. Such a tool can be used to design a novel knowledge-based potential according to the following scheme. Firstly, the repository of descriptors constructed for all residues of nonhomologous proteins from a dataset is created. Next, the descriptors are structurally compared to each other in order to identify descriptor groups, where every descriptor group preserves a unique, conservative 3D shape. Structural alignment of descriptors from each group can be measured with residue-based features (e.g., charge, hydrophobicity) or scores computed by using substitution matrices for sequence and secondary structure. Finally, global or local quality of an input protein 3D structure can be measured as an average of residue scores. Computationally efficient algorithms that provide reliable results of structural comparison of descriptors are a crucial component in the aforementioned puzzle.

We realized the approach by means of combinatorial modeling. We formulated an optimization problem, which is simplified in comparison to the real-world perspective, but it fits requirements regarding constraints and quality of solutions. We proved that the considered problem is equivalent to the assignment problem, and therefore can be solved in polynomial time. These theoretical results are presented in Section Combinatorial model. Three related polynomial-time algorithms based on the Hungarian method were proposed and they are presented together with an exact backtracking algorithm in Section Algorithms. In succeeding sections, their results are compared and discussed. Before that, in the following subsection we describe the problem in detail.

### Description of the problem

A local protein descriptor characterizes a specific structural neighborhood observed around a particular central residue, treated as a center of the descriptor [17]. In principle, the descriptor is represented by a set of discontinuous fragments of a protein chain that are located in the spatial proximity of the central residue. An important advantage of protein descriptors over the continuous fragments of the protein chain is that the descriptors take into consideration long-range (in the sense of amino acid chain) atom-atom interactions that are observed in protein structures. Moreover, structurally similar descriptors can be identified in non-homological proteins. Therefore, protein descriptor conformations are treated as basic, geometrical units of protein folds.

The process of building a descriptor is as follows (see Additional file 1 for the appropriate illustration in Fig. Sf1). In the structural proximity of a central residue, closely located residues are identified (below a given distance threshold). Either central or closely located residues are extended with their neighboring residues in the chain (two adjacent residues on each side) in order to obtain so-called *elements*, which are five-residues-long continuous fragments of the protein backbone. The element constructed for the central residue is called *central*. Elements can overlap and combine together to form a longer continuous fragment called *a segment*. To sum up, the protein descriptor is characterized by a set of elements and segments, with the central element distinguished.

Every *descriptor group* consists of one distinguished protein descriptor, being the group founder, and a set of descriptors that are classified as descriptors structurally similar to the founder. The main advantage of the descriptor group is that it guarantees strict structural mapping between corresponding residues of the group members (a structurally validated alignment of amino acid sequences for the descriptor group from Fig. Sf2 of Additional file 1 is presented in Table 1). Descriptor groups represent different geometrical 3D shapes that are observed in protein structures and can be treated as a specific spatial fingerprint. The groups, constructed from a wide collection of known tertiary structures of proteins, can be used as a structural context in the process of quality assessment [19].

**Table 1:** Example of an alignment of amino acid sequences of protein descriptors from the same group (the group founder is descriptor d1p1da2_A_206_LEU, see also Fig. Sf2 in Additional file 1)

Descriptor name	Segment 1	Segment 2	Segment 3	Segment 4	Segment 5	Segment 6
d1p1da2_A_206_LEU	FHVKLPK	LGITI	DPLVISD	SVAHRTGTLEL	DKLLAIDN	QILQQCEDLVKLKIRK
d1q3oa__A_679_VAL	KTVLLQK	FGFVL	..QYLES	GVAWR.AGLRM	DFLIEVNG	NMIRQ..NTLMVKVVM
d1y7na1_A_84_MET	TTVLIRR	LGFSV	..GIICS	GIAER.GGVRV	HRIIEING	HILSN..GEIHMKTMP
d1x6da1_A_98_ILE	HVTILHK	AGLGF	..ITVHR	GLASQ.GTIQK	NEVLSING	RQARE..RQAVIVTRK
d1v62a__A_96_LEU	..VEIVK	LGISL	..ITIDR	SVVDR.GALHP	DHILSIDG	KLLASISEKVRLEILP
d1w9ea1_A_188_MET	REVILCK	LRLKS	..IFVQL	SPASL.VGLRF	DQVLQING	KVLKQ..EKITMTIRD
d2cssa1_A_110_ILE	GRVILNK	LKVVG	..AFITK	SLADVVGHLRA	DEVLEWNG	NIILE..PQVEIIVSR
d1uf1a__A_98_LEU	KKVNLVL	LTIRG	..IYITG	SEAEG.SGLKV	DQILEVNG	RLLKS..RHLILTVKD

Character “.” means that there is no structural mapping for a particular residue between the founder and the group member

A crucial component of such approach is a precisely constructed structural alignment of protein descriptors. In the literature, slightly different multicriteria functions classifying pairs of protein descriptors as structurally similar were proposed [15, 18]. In principle, all known contact-based functions employ the requirement for a contact between central and other residues, where the contact is defined as distance inequalities for particular atoms. Protein descriptors are described at three levels: segments, elements, and finally particular residues. The measure used in the structural alignment identification process is the *root-mean-square deviation* (RMSD), which is an averaged measure of distance between two sets of corresponding atoms [20]. The RMSD value of 3.5 Å was assumed in the comparison process as an upper bound for treating a pair of protein descriptors as structurally similar [15]. According to RMSD, the compared structures should have the same number of atoms. In general, if there is a need to compare two multi-segment descriptors, which can be composed of different number of atoms, an unambiguous longest structural alignment should be built between them. To simplify the representation of protein descriptors, the segment analysis level is omitted (the lengths of segments often differ).

In the previous studies, the problem of structural alignment of protein descriptors was characterized by a multicriteria assessment function with an asymmetric alignment strategy [14, 15]. Authors introduced the problem as a component of the descriptor libraries generation pipeline, focusing on the biological side of the problem. In this paper, we introduce a mathematical formulation of the descriptors alignment problem together with an efficient algorithmic solution.

## Methods

### Combinatorial model

In our approach, the structural alignment of descriptors is based on the elements analysis level (every element is five residues long). It requires verification of a spatial alignment for all combinations of element pairs identified between compared descriptors, besides central elements. In practice, computational complexity is reduced, because the alignment is constructed on the set of structurally similar duplexes identified in descriptors. A *duplex* is a pair of elements of a descriptor consisting of the central element and one of the others. The presence of the central element in the duplex helps to stabilize such structure in 3D space during the alignment process. Two duplexes are harder to align than aligning two elements alone, because of fewer possibilities of rotating them in the space. The reduction of the complexity results from faster cuts in this process. At the same time, accuracy of the alignment grows. However, we have to be aware that a pair of compared duplexes can differ in the number of residues, when inside a duplex its central element overlaps the other one. Following the work of [15], we classify a descriptor pair as structurally similar if the central elements are preserved (the RMSD value is not greater than 1.2 Å), the resultant alignment is structurally similar (its RMSD value is up to 3.5 Å), and the minimal ratio of elements or residues present in the alignment and descriptors is not less than $\frac {4}{5}$ and $\frac {2}{3}$, respectively.

The answer for the problem introduced in the preceding section can be *the greatest structural alignment* of a pair of compared descriptors A and B (expressed as sets of elements), that is the one involving subsets of their elements of a maximum size, which satisfies the following conditions on descriptors’ similarity.

- Let *A*={*a*_∗_,*a*_1_,…,*a*_*n*_} and *B*={*b*_∗_,*b*_1_,…,*b*_*m*_}, where *a*_∗_ and *b*_∗_ are central elements of the descriptors. The cardinalities of the descriptors must fulfill the inequalities $\frac {4}{5}|B| \le |A| \le \frac {5}{4}|B|$.
- The distance between molecules is expressed by the RMSD measure. This value can be computed only when two compared molecules are composed of the same number of atoms. Let RMSD(*u,v*) be the function returning the RMSD value (expressed in Å) computed for molecules *u* and *v*, or a big value M ≫3.5 if these molecules consist of different number of atoms. In any structural alignment, RMSD(*a*_∗_,*b*_∗_)≤1.2.
- Besides the central elements, the alignment juxtaposes more complex structures, namely duplexes. The sets of duplexes are defined as *D*_*A*_={*d*_*Ai*_=(*a*_∗_,*a*_*i*_) : *i*=1,…,*n*}, *D*_*B*_={*d*_*Bj*_=(*b*_∗_,*b*_*j*_) : *j*=1,…,*m*}, and pairs of elements of these sets are measured by the RMSD function.
- In every structural alignment, the central elements must be involved, and the total number *N* of pairs in the alignment must fulfill the inequalities $N \ge \frac {4}{5}|A|$ and $N \ge \frac {4}{5}|B|$. The alignment must involve at least $\frac {2}{3}$ of the residues of *A* and *B*.
- The RMSD values of all pairs of duplexes in the alignment must not be greater than 3.5 (Å), as well as the global RMSD value computed for the entire aligned substructures of the compared descriptors.

Although RMSD values are (non-negative) real numbers, for our purposes it is enough to round them to one or two decimal positions. The restriction of these values to rational numbers is of importance for further considerations, and therefore we assume that in the remaining of the paper.

A structural alignment of two descriptors, or their fragments, can be accepted in the sense of the RMSD measure only if the numbers of atoms of the two compared structures are equal. If the descriptors are decomposed into series of duplexes, some of them possibly sharing the same residues, the process of merging them partially back can result in substructures of these descriptors, which cannot be evaluated by RMSD. These substructures can have different numbers of residues even if they were built of the same number of duplexes.

Our exact backtracking algorithm, implemented in addition to our main proposition in order to verify its suitability, keeps watching on this condition, and on two supplementary optimization criteria, namely the number of residues in the alignment (maximized) and the average RMSD of aligned duplexes (minimized). Altogether, the problem in such a statement is presumably computationally hard. We propose here another perspective, resulting in a much easier combinatorial model. The model, although being a simplification of the real-world case, is quite satisfying as a close approximation of the former one.

The greatest structural alignment can be modeled as an optimization combinatorial problem, *the maximum-size assignment* (MA), with the following integer linear programming expressions.

$$\begin{array}{*{20}l} \text{maximize} \quad & \sum_{i=1}^{n} \sum_{j=1}^{m} x_{ij}, & & \\ \text{subject} \; \text{to} \quad & \sum_{i=1}^{n} \sum_{j=1}^{m} c_{ij} x_{ij} \le L, & & \\ & \sum_{i=1}^{n} x_{ij} \le 1, & & {\forall}_{j = 1, \dots, m} \\ & \sum_{j=1}^{m} x_{ij} \le 1, & & {\forall}_{i = 1, \dots, n} \\ & x_{ij} \in \{ 0, 1\}, & & {\forall}_{i = 1, \dots, n, \; j = 1, \dots, m} \end{array} $$

where *c*_*ij*_ stands for a cost of aligning objects *i* and *j*, *L* for a limit on total cost of the solution, and *x*_*ij*_ is a decision variable. In our settings, *c*_*ij*_=RMSD(*d*_*Ai*_,*d*_*Bj*_), for *i*=1,…,*n,j*=1,…,*m*, and *L* is a limit set according to a user need. The smaller *L*, the more consistent solution. In the current problem, the number of elements selected to a solution must be similar to the cardinalities of descriptors, therefore for the moment we can assume that *L* depends on the minimal value from the pair *n* and *m* (see Section Algorithms for more precise propositions). The length *N* of the alignment is the value of the maximized function incremented by 1 (1 stands for the central elements of the descriptors).

Not all conditions reported at the beginning of the current section are present in the above formulation; however, they are outside the core problem and can be easily verified either before solving it (the relation between |*A*| and |*B*|, the bounds for RMSD(*a*_∗_,*b*_∗_) and RMSD(*d*_*Ai*_,*d*_*Bj*_)) or after that (the relation between |*A*|, |*B*| and *N*, the bound for the global RMSD of the alignment). The condition for the number of residues in the alignment has been taken out of our model, as having minor significance in the face of the rest, and put at the end of our programs to finally accept (or not) the alignment.

Reduced to the form of MA, the greatest structural alignment resembles the assignment problem or a problem in between the cardinality matching and weighted matching in bipartite graphs (definitions, e.g., in [21]). The former similarity is especially noticeable in the following decision formulations. Without loss of generality, we can assume that all variables are non-negative integers (values in MA multiplied by a constant in order to get rid of rational numbers).

#### **Problem 1.**

Maximum-size assignment problem (*Π*_MA_) — decision version.

- An *n*×*m* matrix *C*=[*c*_*ij*_], bounds *K*≤min{*n,m*} and *L*, all the values being non-negative integers.
- Is there a subset *C*^′^ of the elements in *C*, with at most one element in each row and in each column of *C*, such that |*C*^′^|≥*K* and ${\sum \nolimits }_{c \in C'} c \le L$?

#### **Problem 2.**

Assignment problem (*Π*_A_) — decision version.

- An *n*×*n* matrix *C*=[*c*_*ij*_], bound *L*, all the values being non-negative integers.
- Is there a subset *C*^′^ of the elements in *C*, with exactly one element in each row and in each column of *C*, such that ${\sum \nolimits }_{c \in C'} c \le L$?

These two problems are mutually polynomial-time reducible.

#### **Theorem 1.**

Problems *Π*_MA_ and *Π*_A_ are equivalent.

#### **Proof 1.**

To prove it, we provide two polynomial transformations, from *Π*_MA_ to *Π*_A_ and from *Π*_A_ to *Π*_MA_, and, in both cases, we show that every instance of one problem gives the answer “yes” if and only if the transformed instance of the other problem gives the answer “yes”.

The transformation of *Π*_MA_ to *Π*_A_ is defined as follows. Create square matrix *C*_A_ on the basis of *C*_MA_ by adding rows and columns filled by big values M>*L*_MA_ or zeroes, in the following way. Set *n*_A_=*n*_MA_+*m*_MA_−*K*_MA_. Copy *C*_MA_ to the sector lying at the intersection of first *n*_MA_ rows and first *m*_MA_ columns of *C*_A_. Assign 0 to every entry at the intersection of first *n*_MA_ rows and last *n*_MA_−*K*_MA_ columns, and last *m*_MA_−*K*_MA_ rows and first *m*_MA_ columns of *C*_A_. Set all remaining entries of *C*_A_ (located at the intersection of last *m*_MA_−*K*_MA_ rows and last *n*_MA_−*K*_MA_ columns) to M. Let *L*_A_=*L*_MA_.

Let us assume that an instance of *Π*_MA_ gives the positive answer. It means that the solution $C^{\prime }_{\text {MA}}$ is composed of at least *K*_MA_ elements summing up to a value that is not greater than *L*_MA_. Then, the solution $C^{\prime }_{\mathrm {A}}$ of *Π*_A_ in the instance after the transformation can be constructed by choosing any *K*_MA_ elements from $C^{\prime }_{\text {MA}}$ and complementing them with elements from not-yet-involved first *n*_MA_ rows and first *m*_MA_ columns of *C*_A_, precisely from their last *n*_MA_−*K*_MA_ or *m*_MA_−*K*_MA_ entries, respectively (these elements are zeroes). We always have *n*_MA_−*K*_MA_ rows (among the first *n*_MA_ rows of *C*_A_) and *m*_MA_−*K*_MA_ columns (among the first *m*_MA_ columns of *C*_A_), which are not involved in the initial solution of cardinality *K*_MA_. Therefore, these rows/columns bind all columns/rows added to *C*_MA_ during the transformation (each one bound up exactly once). All rows and columns of *C*_A_ are now represented in $C^{\prime }_{\mathrm {A}}$ and none of values M is used. Since every element *c*∈*C*A′∖*C*MA′ is equal to 0, the sum of elements from $C^{\prime }_{\mathrm {A}}$ cannot be greater than *L*_A_. The answer for *Π*_A_ is “yes”.

Now let us assume that an instance of *Π*_A_ (after the transformation) gives the positive answer. Then, $C^{\prime }_{\mathrm {A}}$ is composed of *n*_A_ elements summing up to a value not greater than *L*_A_, the elements located in distinct rows and columns of *C*_A_. None of these elements can be equal to M, therefore at least *n*_MA_+*m*_MA_−2*K*_MA_=*n*_A_−*K*_MA_ elements are equal to 0. The sum of the remaining *K*_MA_ elements still has *L*_A_=*L*_MA_ as the upper bound, and these elements are located at the intersection of first *n*_MA_ rows and first *m*_MA_ columns of *C*_A_. Since the mentioned sector covers matrix *C*_MA_, these *K*_MA_ elements compose a proper solution of MA and we get the answer “yes” for the decision problem *Π*_MA_.

Given an instance of *Π*_A_, the construction of the corresponding instance of *Π*_MA_ is straightforward. Let *n*_MA_=*m*_MA_=*K*_MA_=*n*_A_, *C*_MA_=*C*_A_, and *L*_MA_=*L*_A_.

Let an instance of *Π*_A_ give the positive answer. We then have *n*_A_ elements, every element located in a different row and column of *C*_A_, summing up to at most *L*_A_. It is also a proper solution for *Π*_MA_, and therefore the answer for the latter problem is also positive.

In the other case, if an instance of *Π*_MA_ (after the transformation) gives the answer “yes”, the solution must also satisfy conditions for $C^{\prime }_{\mathrm {A}}$. The cardinality of $C^{\prime }_{\text {MA}}$ is equal to *n*_A_, and therefore all its elements must be located in distinct rows and columns of *C*_MA_ and all rows and columns are occupied. The limits on their sums are the same, therefore the answer for *Π*_A_ is “yes”. □

The assignment problem can be solved in O(*n*^3^) time by the Hungarian algorithm [22, 23] or by a reduction to the min-cost flow problem and searching for augmenting paths [21]. The proof gives us a title to solve the greatest structural alignment problem by these means. As shown in the following paragraph, it can be done in O(*n*^4^) time.

### Algorithms

In the following algorithms, we use the Hungarian method as a subroutine, which returns an optimal assignment for a current number *K* of pairs of objects. In the construction of the input for the Hungarian method, we transform matrix *C*_MA_ to *C*_A_ as in the proof of the theorem above. Because *K* is not explicitly given in the optimization version of the problem, the method is executed, in turn, for *K* taking values from the greatest possible, i.e., min(*n,m*), to the lowest one satisfying the bound for *N* defined at the beginning of the previous section, i.e., $\lceil \frac {4}{5}(\max (n,m)+1)\rceil -1$. The number of these iterations can be reduced: firstly by stopping at such *K* that satisfies all constraints; and secondly by extracting a feasible subsolution from a non-feasible solution obtained for a greater *K*. The latter is incorrect in general, however it can be applied only when the subsolution is accompanied by several big values M, and no other values are present in the larger solution.

Such application of the Hungarian method ensures that, besides the global criterion of the maximum-size assignment problem (the size of the assignment), we realize a secondary criterion, which is the total cost of the solution being minimized. For a given *K*, we obtain the cheapest assignment, what is important from the biological point of view. Although all solutions of costs below the bound *L* are probable to form an acceptable structural alignment (with regard to the global RMSD), the lower the cost, the higher the probability.

With this approach we have solved also another issue, since we could set up the value of *L* more precisely than basing solely on *n* and *m*. Currently, we assign to *L* the product of *K* and a factor *f*. Setting *f* to 3.5 makes the constraint for the total cost nearly useless (except rejecting Ms), because all pairs of duplexes considered in a feasible solution have their RMSD values at most 3.5 (greater costs in *C*_MA_ can be switched to M). On the other hand, the lower *f*, the greater the number of omitted feasible solutions. A drawback of the minimum-size assignment problem, in comparison to the real-world model applied in our backtracking algorithm, is that we get here one optimal solution only. Even though it is most probable to form an acceptable structural alignment, it might not do it (if the number of atoms in the aligned structures are different), while the backtracking algorithm explores all feasible solutions and gives certainty about feasibility and optimality of the output. In our study $f \in \langle \frac {1}{2}\cdot 3.5; \frac {2}{3}\cdot 3.5 \rangle $.

#### **Example 1.**

**Fig. 1:**
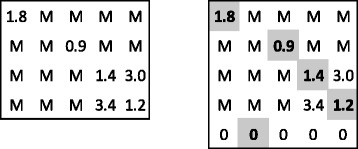
A cost matrix *C*_MA_ (on the left) and the corresponding matrix *C*_A_ (on the right). The solution of the assignment problem is denoted with the gray background

Let descriptor *A* be composed of the central element and four others, and *B* of its central element and five others. Therefore, *n*=4, *m*=5, and *N* could be equal only to 5 (i.e., *K*=4), if any feasible alignment exists. Let *L*=2*K* and the cost matrix *C*, filled by RMSD values for the appropriate pairs of duplexes, be as in the left part of Fig. 1. Obviously, we assume here that RMSD(*a*_∗_,*b*_∗_)≤1.2.

The Hungarian method is executed with the input matrix as in the right part of Fig. 1. The solution satisfies the constraint for *L*, therefore it is a correct solution for the maximum-size assignment problem. For verifying feasibility for the problem of greatest structural alignment, the alignment of the entire solution measured by the RMSD function cannot exceed 3.5.

**Fig. 2:**
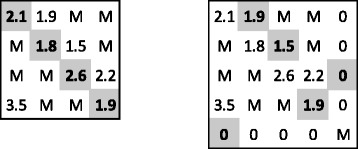
A matrix *C*_MA_=*C*_A_ for *K*=4 (on the left) and another *C*_A_ for the same *C*_MA_ and *K*=3 (on the right). The solutions of the assignment problem are denoted with the gray background

Now take another cost matrix, the one presented in Fig. 2 (on the left). We see that *n*=*m*=4 and *K* belongs to the interval 〈3,4〉. Therefore, *N* can be equal to 4 or 5, and *L*=2*K* is equal to 6 or 8, respectively. First, the Hungarian method is executed for *K*=4 and for the same cost matrix as in the original problem. The solution (on the gray background, in the left part of Fig. 2) is not a feasible one of the maximum-size assignment problem, because its cost is greater than 8. For *K*=3, the cost matrix for the Hungarian method is presented on the right in Fig. 2. These assigned pairs (on the gray background), which are located at the intersection of first *n* rows and first *m* columns, constitute the solution of the maximum-size assignment problem, because sum of their costs is not greater than 6. Finally, for the solution its global RMSD is computed and compared with the bound.

First of the proposed algorithms solves the greatest structural alignment problem as sketched above. Next two ones are supplemented by additional operations performed at the stage of looking for a solution, which allow choosing more promising paths in the solution space. These additional steps are still executed in polynomial time. All the algorithms are just parts of the entire process for solving the biological problem; other parts enclose procedures for browsing the library of descriptor sets, verifying the number of residues composing the alignment or summarizing results.

#### **Algorithm 1.**

The maximum-size assignment problem is being solved in the loop with decreasing value of *K*, and the first feasible solution of the problem is optimal and taken as the answer of this stage. Next, the global RMSD is computed for the alignment of protein substructures composed of pairs of duplexes from the assignment supplemented by the pair of central elements of the descriptors. When the global RMSD value is not greater than 3.5 Å the solution is returned as the output of the algorithm, otherwise no solution has been found.

#### **Algorithm 2.**

The maximum-size assignment problem is solved for all values of *K* from the given range, all obtained solutions of the problem are remembered on a list. Next, for every entry of the list the global RMSD is computed and among those satisfying this constraint the one that is composed of the greatest number of duplexes (the primary optimization criterion) and the greatest number of residues (the secondary optimization criterion) is chosen as the answer of the algorithm. If no one satisfies the bound for the global RMSD, no solution is returned as the output.

#### **Algorithm 3.**

The maximum-size assignment problem is solved for all values of *K* from the given range. In addition to all solutions, also all feasible partial solutions are stored on a list. A partial solution is based on a prefix of the final solution obtained for a given *K*, where the latter has the form of the list of assigned pairs of duplexes sorted by their costs in the non-decreasing order. All sufficiently long prefixes are considered. A partial solution is created by adding one by one, in the order, these pairs from a prefix, which jointly satisfy the condition on an equal number of residues in the two aligned substructures of descriptors. If some of the pairs of the prefix make the formed partial solution unacceptable (because of different number of residues), they are omitted. A partial solution is feasible and remembered for further consideration, if it satisfies the bound for *L* from the formulation of the maximum-size assignment problem and the bound for *K*.

After that, the bound for the global RMSD is verified for every entry of the list of solutions and partial solutions. Among the items satisfying this constraint, the one that is composed of, first, the greatest number of duplexes and next, the greatest number of residues is chosen as the answer of the algorithm.

Additionally, as a reference in an analysis of results of the computational experiment, we propose a new exact backtracking algorithm, which solves the real-world version of the biological problem in exponential time.

#### **Algorithm 4.**

In comparison to the combinatorial formulation held in Algorithms 1–3, the exact algorithm realizes additional optimization criteria during construction of solutions and examines the entire solution space. These supplementary criteria are: the number of residues in the alignment (maximized) and the average RMSD of aligned duplexes (minimized). The algorithm identifies the greatest alignment according to the number of included duplexes (the primary criterion), which has the average RMSD value of these duplexes as low as possible (the secondary criterion). Moreover, among alignments composed of an equal number of duplexes, the ones that cover a greater number of residues are preferred. The feasibility of potential solutions is verified from the point of view of the constraints for the global RMSD of the aligned substructures, the number of covered duplexes and residues.

## Results

All the algorithms described in the previous section were developed in Java and integrated in a computationally efficient tool descs-standalone, which allows a user to identify and structurally compare descriptors of biological molecules, such as proteins and RNAs. The most important advantages of the proposed approach are the following: (1) a flexible representation of an expression used for identification of in-contact residues located in the proximity of the descriptor’s center that can be simply introduced by a user; (2) an application of the BioJava framework [24], which ensures a consistent representation of 3D structures of biological molecules in two formats, PDB and mmCIF; and (3) a publication of the tool as an open-source project available at GitHub (https://github.com/mantczak/descs-standalone).

We performed computational experiment with real-world biological data to verify efficiency of the proposed algorithms. The evaluation process was conducted on representative descriptor sets that were retrieved randomly from ASTRAL 1.75A compendium of 3D structures of protein domains [25]. At the stage of identifying descriptors, residues close to the center of a particular descriptor were selected with the following expression [15]:

OR(DISTANCE:SCGC ≤6.5, AND(DISTANCE:SCGC ≤DISTANCE:CA −0.75, DISTANCE:SCGC ≤8.0)).

We decided to use C _*α*_ (CA) and the geometrical center of side-chain (SCGC) as representative atoms for every residue of a descriptor. The dataset was divided into subsets due to the number of descriptor elements, between 3 and 11. All pairs of descriptors within a set were structurally compared. Cardinalities of the considered descriptor sets and numbers of descriptor pairs, which have been classified as structurally similar by the exact backtracking algorithm (i.e., Algorithm 4) and included in the *reference set*, used during verification of reliability of other proposed algorithms, are shown in Table 2. Tests were performed on a single processor, Intel Core i7 2.66 GHz, HT, 8 GB, under Ubuntu 12.04.

**Table 2:** The dataset used in the experiment of the structural comparison of descriptors

Descriptor elements count	Considered descriptors count	All similar descriptor pairs count
3	1657	340
4	1631	100
5	1590	238
6	1544	144
7	1494	109
8	1446	117
9	1400	203
10	1346	350
11	1301	421

Table 3 presents average processing times of the algorithms, in milliseconds. The average values have been computed for these instances, which were classified as structurally similar by Algorithm 4. As one could foresee, times for the polynomial-time algorithms are much more stable, as they are accompanied by low standard deviation values. Times of the exact algorithm are relatively low, as long as too large sizes of the instances are not considered.

**Table 3:** Summary of processing time [ms]

	Algorithm 1		Algorithm 2		Algorithm 3		Algorithm 4	
Descriptor elements count	avg.	std. dev.	avg.	std. dev.	avg.	std. dev.	avg.	std. dev.
3	4.3	0.5	7.8	0.5	9.4	0.5	5.3	0.5
4	5.3	0.6	8.9	0.7	15.1	0.9	7.0	1.3
5	5.1	0.8	8.0	1.8	20.5	4.9	13.3	11.2
6	5.9	0.9	12.4	2.3	39.5	6.1	42.2	48.6
7	6.1	0.9	14.0	2.5	61.5	6.8	97.0	124.6
8	6.2	0.9	14.8	2.8	91.1	7.5	448.8	372.2
9	6.0	1.1	16.5	3.5	109.8	7.6	2743.7	3362.4
10	5.9	1.1	16.2	4.0	121.8	9.7	12267.0	21504.2
11	6.6	1.3	28.5	7.4	140.6	8.3	256785.6	424309.9

Summary of quality of results obtained for all instance sets (descriptor sizes 3–11) is presented in Table 4. For each of Algorithms 1–3, three values of threshold *f* were considered (1.75, 2.0, 2.33), where *f* is a factor, which together with *K* defines the limit *L* for the total cost of feasible solutions (see Section Algorithms for explanation). Detailed results are placed in Additional file 1 (Tables St1–St3), here mean values are presented, and each cell contains an average computed for one column of the appropriate table from the supplement. The presented data allow us to answer to the following questions: How often are the heuristics able to find an optimal solution? How does a feasible solution differ from the optimal one with regard to quality of structural alignment when an optimal solution is not found? To answer the first question we have measured *coverage of similar descriptor pairs*, which denotes, in percentage points, the ratio of a number of descriptor pairs, which have been classified as structurally similar by the given algorithm to a cardinality of the corresponding reference set. To find an answer to the more specific question we have analyzed measures, which allow us to assess precisely a difference between the heuristic and exact solutions, namely the ratio of aligned residues to all residues in a descriptor and the global RMSD score (see Section Description of the problem for explanation) computed for the corresponding sets of representative atoms of aligned residues. We have assumed that structural alignments obtained by two different algorithms are indistinguishable from the quality point of view when they are characterized with the same values of both measures. Therefore, *quality identity* denotes this fraction of the descriptor pairs that were classified as structurally similar by the given algorithm, for which the solution is indistinguishable from the quality point of view from the optimal one. Next two columns describe the cases when solutions differ in quality. All solutions given by the algorithm that have the same value of the aligned residues ratio as the optimal solution, but are characterized by a higher global RMSD value than the optimal counterpart, are counted in column *higher global RMSD, equal residues ratio*. Such solutions are almost as good as the optimal ones. In column *lower residues ratio* all remaining solutions are counted. In every row of the table, values in the cells *quality identity*, *higher global RMSD, equal residues ratio*, and *lower residues ratio* sum up to 100 %. The higher residues ratio or the lower global RMSD for an equal value of the residues ratio, the better alignment, however, every alignment enclosed here has been finally accepted due to all the considered problem constraints.

**Table 4:** Summary of solutions quality (for elements count in the range between 3 and 11)

Algorithm (threshold)	Coverage of similar descriptor pairs [%]	Quality identity [%]	Higher gl. RMSD, equal resid. ratio [%]	Lower residues ratio [%]	Global RMSD [Å]
1 (1.75)	70.60	96.43	1.42	2.15	2.01
1 (2.0)	77.54	97.66	1.43	0.91	2.12
1 (2.33)	81.90	98.44	1.32	0.23	2.18
2 (1.75)	75.19	95.45	1.75	2.80	2.00
2 (2.0)	85.59	96.02	1.88	2.10	2.12
2 (2.33)	91.35	96.44	1.95	1.61	2.20
3 (1.75)	80.41	92.10	3.31	4.59	2.05
3 (2.0)	87.92	94.36	3.10	2.54	2.14
3 (2.33)	93.07	95.00	3.06	1.94	2.21
4	100.00	100.00	0.00	0.00	2.28

Among the detailed results in Tables St1–St3 we can see that the outcomes of Algorithms 1–3 are significantly worse for the smallest descriptors, i.e., the ones of sizes 3 and 4. This fact follows the combinatorial model, where for such descriptors only one solution is further verified, regardless of the version of the polynomial-time algorithm. The only possibility is to align two complete sets of duplexes of compared descriptors. The situation can also be explained from the biological point of view. Such small descriptors are rather located on a surface of a protein (being very flexible) and therefore hard to be aligned to other descriptors. Taking this into consideration, we have prepared Table 5, where the results are truncated to the descriptor sets of sizes 5–11.

**Table 5:** Summary of solutions quality (for elements count in the range between 5 and 11)

Algorithm (threshold)	Coverage of similar descriptor pairs [%]	Quality identity [%]	Higher gl. RMSD, equal resid. ratio [%]	Lower residues ratio [%]	Global RMSD [Å]
1 (1.75)	78.13	95.40	1.83	2.77	2.12
1 (2.0)	82.64	97.00	1.84	1.16	2.20
1 (2.33)	82.03	98.00	1.70	0.30	2.21
2 (1.75)	84.04	94.16	2.25	3.60	2.11
2 (2.0)	92.99	94.88	2.42	2.70	2.21
2 (2.33)	94.18	95.42	2.51	2.07	2.22
3 (1.75)	90.75	89.85	4.26	5.90	2.17
3 (2.0)	95.99	92.74	3.98	3.27	2.24
3 (2.33)	96.40	93.57	3.94	2.49	2.25
4	100.00	100.00	0.00	0.00	2.27

In Additional file 1, a few example instances of the problem and the optimal solutions are presented, see Fig. Sf3.

From the application point of view, our tool is the first freely available software package that allows for identification and comprehensive analysis of residue-residue contacts-driven structural motifs of protein 3D structures based on the concept of descriptor. The most important advantage is a flexibility that allows the user to do the following: to set the size of an element, to apply own expression used for the identification of close residues in the structural proximity of a descriptor’s center, and to set values of thresholds controlling the process of structural comparison.

In the descriptors *identification mode*, the user sets the following input parameters: (1) a 3D structure of a protein in one of the supported formats, PDB or mmCIF (by default the PDB format is assumed); (2) the molecule type (because the tool supports also RNAs); and (3) an expression that is used for the identification of residues closely located around the descriptor’s center. As a result, the set of descriptors is stored in the output directory. In general, for every proper residue (i.e., complete set of atom coordinates, unmodified residues) of the input protein 3D structure, one descriptor is built and stored in a PDB file. A path for the output directory and the output format can be set by the user. It is worth to mention that the element size, which is by default equal to 5, can also be defined as other natural odd number. Moreover, there is also a possibility to store only specific kinds of descriptors (e.g., when they consist of three or more segments) using thresholds, set by the user, associated with the number of segments, elements, and residues. A flexible representation of the expression used for identification of in-contact residues supports the following basic operators: logical (OR, AND, NOT), relational (<,≤,=,≥,>), and arithmetic ones. The user can use DISTANCE operator between any pair of atoms, except hydrogen that are included in the 3D structure of the input molecule (e.g., DISTANCE:CA;CB, DISTANCE:CA). Moreover, a few virtual atoms can also be applied in the expression, e.g., as a representative of the side-chain of a residue either C _*β*_-extended point (CBX) [15] or geometrical center of side-chain (SCGC), when the user needs to use the same point in space relative to the backbone independently from the residue type or different points in space directly dependent on the residue type, respectively.

In the *mode of structural comparison* of a descriptor pair, the user sets the following input parameters: (1) 3D structures of a pair of descriptors that is to be compared; (2) the selected comparison algorithm; when the Hungarian method-based algorithms are applied, there is also a need to set the value of the threshold parameter *f*, by default equal to 2.33, which is the maximal allowed RMSD-based cost of a pair of aligned duplexes; (3) atom names of the residue representatives that have been used during the identification of descriptors and should be considered during the construction of the resultant structural alignment; and (4) the output directory path. As a result, the analyzed pair of descriptors is structurally aligned and classified as structurally similar or not, if their greatest alignment satisfies the criteria explained in Section Combinatorial model. Values of thresholds set for the considered criteria can be treated as a proposition, and therefore can be simply modified to meet the user expectations. Every resultant structural alignment is analyzed with the measures such as the ratio of aligned elements (or residues) to all elements (or all residues) in a descriptor and the global RMSD value which is computed for the corresponding sets of representative atoms of aligned residues. These scores are also presented to the user. Moreover, the result of structural comparison can be supplemented with aligned 3D structures of compared descriptors stored in format PDB (or mmCIF) on user demand. We want to emphasize that a descriptor pair can be structurally compared only if both descriptors were built with the same size of the element.

A detailed list of provided options and usage scenario examples prepared for all provided execution modes are available at the project web page http://www.cs.put.poznan.pl/mantczak/index.php?slab=descs-standalone in sections “Execution modes” and “How to run descs-standalone”, respectively.

## Discussion

Algorithm 1 is a straightforward implementation of the combinatorial approach reducing the biological problem of the structural alignment of descriptors to the maximum-size assignment problem. It is a simplification of the real-world case, but resulting in quite satisfying accuracy, when we take into account its low computational complexity and only one alignment produced before the stage of final verification of its feasibility. In comparison to the exact, exponential-time backtracking algorithm it hits 81.9 % of the descriptors classified as structurally similar (for *f*=2.33), where the value is computed as the average for all mean coverages obtained for considered sets of descriptors of particular sizes.

Algorithm 2 differs from Algorithm 1 only in the detail that it produces a few potential alignments, one for each acceptable size *K* from a predefined range. The range is very narrow because for the smallest descriptors (sizes 3 and 4) only one value of parameter *K*, and for the biggest ones (sizes 10 and 11) three values of parameter *K* are considered. Thus, we obtain only one, two, or three potential alignments, later selected according to their feasibility. Such a little modification has allowed for a significant improvement of results, because the set of descriptor pairs qualified as structurally similar by the algorithm has grown, on average, to 91.4 % (for *f*=2.33).

The third algorithm built upon the proposed combinatorial model is the most enhanced and quality of its results is the highest. The percentage of hits to the set of optimal solutions of similar descriptor pairs, computed as above, is 93.1 % (for *f*=2.33). It is also better than previously mentioned algorithms for lower values of parameter *f*, and the difference in the mean coverage is even better noticeable.

The lower the value of *f*, the more restrictive selection, and for a certain *K* the higher probability that the solution will not be found. Therefore, a given pair of compared descriptors has a greater chance to be not classified as structurally similar. However, there is a need to set a low value of *f* in such cases, when one wants to tighten criteria and approve descriptor pairs, whose structural alignment is better than usual. The sense of a lower value of *f* is better noticeable for descriptors of a greater size. Thanks to a more stringent limit, greater alignments will be refused in favor of smaller ones, but the latter more likely fulfill the bound for the global RMSD. Unfortunately, the smallest descriptor sizes 3 and 4 do not allow for decreasing *K* and for them a lower value of *f* is rather not helpful (see detailed results in Additional file 1).

Algorithm 4 copes perfectly with sizes 3 and 4, because it explores the entire solution space and does not confine to one likely alignment. Algorithms 1–3 produce for such descriptors only one solution, which is later verified and, unfortunately often, refused. However, both kinds of the proposed approaches, the exact and polynomial-time ones, complement each other and are convenient to be applied for different ranges of sizes.

Processing efficiency of Algorithm 4 is quite satisfying, when we take into account its computational complexity. For the greatest descriptors (of size 11), it works ca. 424 s per positively classified instance, on average. However, the total time necessary for processing the entire descriptors set becomes huge. It becomes significant even for smaller sizes, for example 7, where processing all pairs within the set of cardinality 1494 takes almost two hours. When one considers analyzing all the descriptor sets at once, especially if the descriptor size can reach even 17, or protein domain libraries are broader than used here, the polynomial-time algorithms are the only option.

The main advantage of the heuristics, based on the combinatorial model, over the exact backtracking algorithm is significantly shorter processing time needed to achieve at least comparable and often indistinguishable results in terms of accuracy. These can be observed in the summary of results, obtained for top 10 of computationally expensive descriptor pairs, presented in Table St4. All considered descriptors were composed of 11 elements; therefore, they belong to the most structurally complex group, thus the processing time of the exact algorithm was significant. For 9 of 10 descriptor pairs, at least one of the proposed heuristics found the optimal solution. Variability within the algorithms’ output presented in the table is almost unnoticeable. Therefore, we present in Table St5 most often changing results, in the sense of the aligned residues ratio, for descriptors of different sizes. These results also allow for observing that the proposed heuristics are computationally efficient, especially for greater descriptors. The problem with finding a solution is observed for Hungarian method-driven algorithms if *f*=1.75. Detailed information about the greatest pair of descriptors from Table St5 is presented in Table St9. One can find there not only measurable features of the compared descriptors, but also their structural alignments obtained as results of Algorithms 1–4. Visualization of these structural alignments is presented in Fig. Sf7. As one can see, all the heuristic algorithms, although more or less advanced and differ in the output, produce valuable alignments very similar to the optimal solution.

In general, a protein descriptor is a short, discontinuous fragment of a polypeptide chain. Therefore, to solve the problem of structural comparison of descriptors one could use a general purpose method for solving the structural comparison of proteins. However, such a method should provide at the output an alignment constructed for a pair of protein descriptors in a form of unambiguous mapping of aligned residues rather than only a single similarity score. Moreover, a feasibility of the resultant alignment should be verified in the sense of ensuring that all the constraints specified in the problem definition of the structural comparison of descriptors resulting directly from their spatial topology are satisfied. Namely, (1) central elements are properly aligned if their residues are aligned exactly to each other (both central elements cannot be shorter and cannot be aligned to other fragments of the descriptors except themselves), (2) residues are properly aligned only when come from fully represented elements (i.e., if a particular element consists of five residues, then all of them must be included in the properly constructed alignment) and structurally similar duplexes (i.e., the RMSD value computed for them cannot be greater than 3.5 Å). Such an unambiguous mapping of aligned residues is provided by DEDAL [18], which is a web server designed for solving the protein structure alignment problem. This is a computationally efficient general purpose method that is driven by the structural comparison of protein descriptors. Therefore, we performed tests to prepare a comparison of the proposed algorithms with this tool, for our dataset of all 2022 structurally similar descriptor pairs. All alignments in which the residues included in the central elements were not properly aligned were discarded. All other improperly aligned residues were filtered out but the alignments obtained in such a way were considered for further analysis. Among all considered descriptor pairs, we found 1.93 % and 60.93 % cases in which the resultant alignment was not provided and was discarded as infeasible, respectively. For the rest, 37.14 %, the obtained alignment satisfied all the constraints specified in the problem definition of the structural comparison of descriptors. Within the latter set, for 69.24 % of pairs DEDAL found the optimal alignment, for 1.60 % of pairs the alignment length was also optimal (in the sense of aligned residues) but its RMSD value was slightly higher, and for 29.16 % of pairs the resultant alignment was shorter in comparison with the optimal counterpart. It must be stressed, however, that such a comparison provides only a rough view, because a general purpose method designed for solving the problem of the structural comparison of proteins cannot be aware of specific constraints resulting from spatial topology of descriptors that are crucial in the problem definition of the structural comparison of protein descriptors.

To demonstrate the flexibility of the tool in the identification mode, we did additional tests with changing expressions and element sizes. For two example residues, A123-VAL (d1e0ta1) and A30-PHE (d2f5ya1), structural motifs in their proximity were built with the element size equal to 3, 5, or 7. Common types of expressions for the identification of in-contact residues were chosen:

OR(DISTANCE:CBX ≤ 6.5, AND(DISTANCE:CBX ≤ DISTANCE:CA − 0.75, DISTANCE:CBX ≤ 8.0))

and

OR(DISTANCE:SCGC ≤ 6.5, AND(DISTANCE:SCGC ≤ DISTANCE:CA − 0.75, DISTANCE:SCGC ≤ 8.0)).

A spatial environment observed around these residues is interesting from the structural topology point of view, therefore allows for presenting diversity of 3D shapes that can be obtained through slight changes of values of input parameters. Visualization of 3D structures of the structural motifs constructed in the proximity of residue either A123-VAL (d1e0ta1) or A30-PHE (d2f5ya1) is presented in Figs. Sf4 and Sf5, respectively. Measurable features, such as numbers of segments, elements, and residues belonging to these structural motifs as well as their sequences are presented in Tables St6 and St7. As we can see, structural motifs constructed with the element size equal to 3 are generally too small to cover secondary structures. The number of segments belonging to these motifs is inversely correlated with the element size. Motifs built with the expression, where C _*β*_-extended point is used as a representative of the side-chain, are more general from definition, therefore more often can be identified even in nonhomologous proteins. Motifs constructed with the use of the geometrical center of side-chain are more specific, harder to identify in various protein 3D structures, and therefore they seem to be more promising to apply in a method for the single mode assessment. It should be emphasized that the tool provided here is highly configurable and allows the user to define own conditions that meet his expectations.

The last tests were based on various expressions for identification of in-contact residues published in the literature, which are as follows:

DISTANCE:CBX < 6.5 [14,17],

OR(DISTANCE:CBX < 6.5, AND(DISTANCE:CBX < DISTANCE:CA − 0.75, DISTANCE:CBX < 8.0)) [15],

OR(DISTANCE:CA ≤ 6.5, AND(DISTANCE:SCGC ≤ DISTANCE:CA − 0.75, DISTANCE:SCGC ≤ 8.0)) [18].

Descriptors for the tests were generated in the proximity of the residues analyzed previously supplemented with A2309-VAL (d2w0pa1). In Table St8, detailed information about these descriptors is presented, and visualization of their 3D structures is in Fig. Sf6.

## Conclusions

The formulation of the structural alignment of protein descriptors was present in the literature only in the form of a general description and a multicriteria assessment function. Here, the problem has been analyzed from the algorithmic point of view more deeply. It has been reduced to the combinatorial model based on the maximum-size assignment problem and solved by polynomial-time algorithms in three versions. Moreover, an exponential-time backtracking algorithm has been proposed to generate solutions that satisfy all requirements of biologists’ practice. Results of all proposed algorithms obtained for real-world biological data have been compared and discussed. It is worth to mention that the algorithms assure symmetry in the process of the structural alignment of descriptors (i.e., matching descriptor A to descriptor B gives the same result as matching B to A).

The combinatorial model, although being a simplification in comparison to reality, has proved to be relevant from the point of view of accuracy of results. Its processing time is an unquestionable advantage over the backtracking algorithm. The latter, in contrast, wins by exploring the entire solution space. Both approaches complement each other as they might be used for descriptor sizes belonging to different ranges.

Proposed methods can be successfully applied in the process of protein 3D model quality assessment. As a large number of comparisons must be done there, the methods will make the process significantly more efficient. With the proposed programs, new libraries of protein descriptors can also be determined and applied during protein 3D structure modeling experiments. The provided tool is the first freely available software package that allows for identification and comprehensive analysis of residue-residue contacts-driven structural motifs of protein 3D structures based on the concept of descriptor.

As a future work, we plan to develop our ideas on the ground of RNA structures comparison. As the opposite to our former method, RNAssess [26], which is used to assess quality of a 3D model in comparison with a reference structure, we will apply a concept of local descriptors in order to solve a harder problem, namely to develop a reliable, general quality assessment method dedicated for RNAs, when the reference structure is not known. Moreover, we are also interested in the analysis of long-range interactions occurring in protein-RNA complexes. We would like to identify libraries of conservative structural motifs that affect significantly on the function of molecular complexes. We hope that the proposed algorithms can be successfully applied in the new contexts.

## Additional file

Additional file 1 Supplementary data. The supplement contains the following: a scheme of building a protein descriptor, visualization of a descriptor group, visualization of solutions of the problem of structural alignment of descriptors generated by the proposed algorithms, visualization of structural motifs constructed with different input parameters, and tables with detailed computational results. All molecular drawings have been prepared with PyMOL (www.pymol.org). (PDF 12,902 kb)

## Abbreviations

CA: Atom C _*α*_
CB: Atom C _*β*_
CBX: C _*β*_-extended point
DNA: Deoxyribonucleic acid
MA: Maximum-size assignment problem
mmCIF: Macromolecular crystallographic information file
PDB: Protein Data Bank
RMSD: Root-mean-square deviation
RNA: Ribonucleic acid
SCGC: Geometrical center of side-chain
