# Supplementary material for: Structural alignment of protein descriptors – a combinatorial model
Source: BMC Bioinformatics. 2016 Sep 17;17:383. doi: 10.1186/s12859-016-1237-9 (PMC5027075; doi:10.1186/s12859-016-1237-9)
Supplement: Additional file 1 — Supplementary data. The supplement contains the following: a scheme of building a protein descriptor, visualization of a descriptor group, visualization of solutions of the problem of structural alignment of descriptors generated by the proposed algorithms, visualization of structural motifs constructed with different input parameters, and tables with detailed computational results. All molecular drawings have been prepared with PyMOL (www.pymol.org). (PDF 12,902 kb) [file 12859_2016_1237_MOESM1_ESM.pdf]

1 Supplementary figures

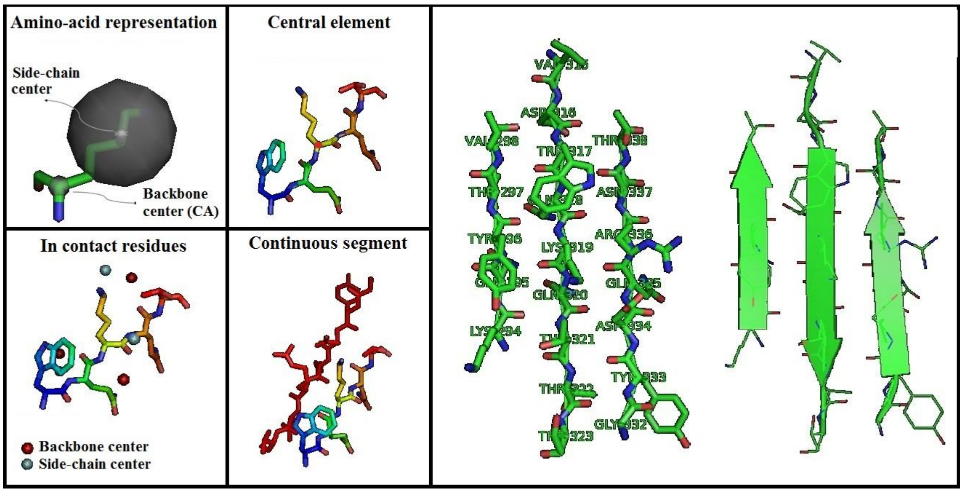

Figure Sf1: A scheme of building a protein descriptor

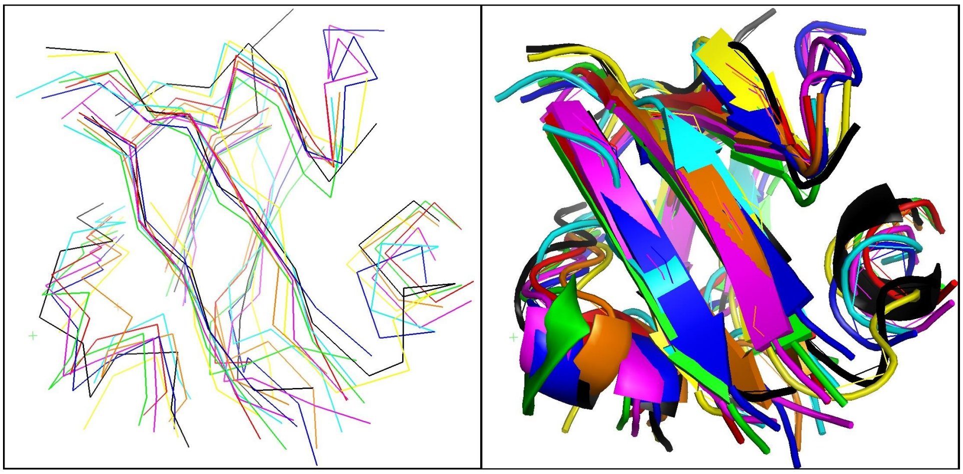

Figure Sf2: Visualization of an example group of descriptors. The group consists of eight descriptors including the founder, which is marked with black. The visualization of a superposition of descriptors is presented in the following views: the descriptor backbones only (on the left) and the cartoon view (on the right)

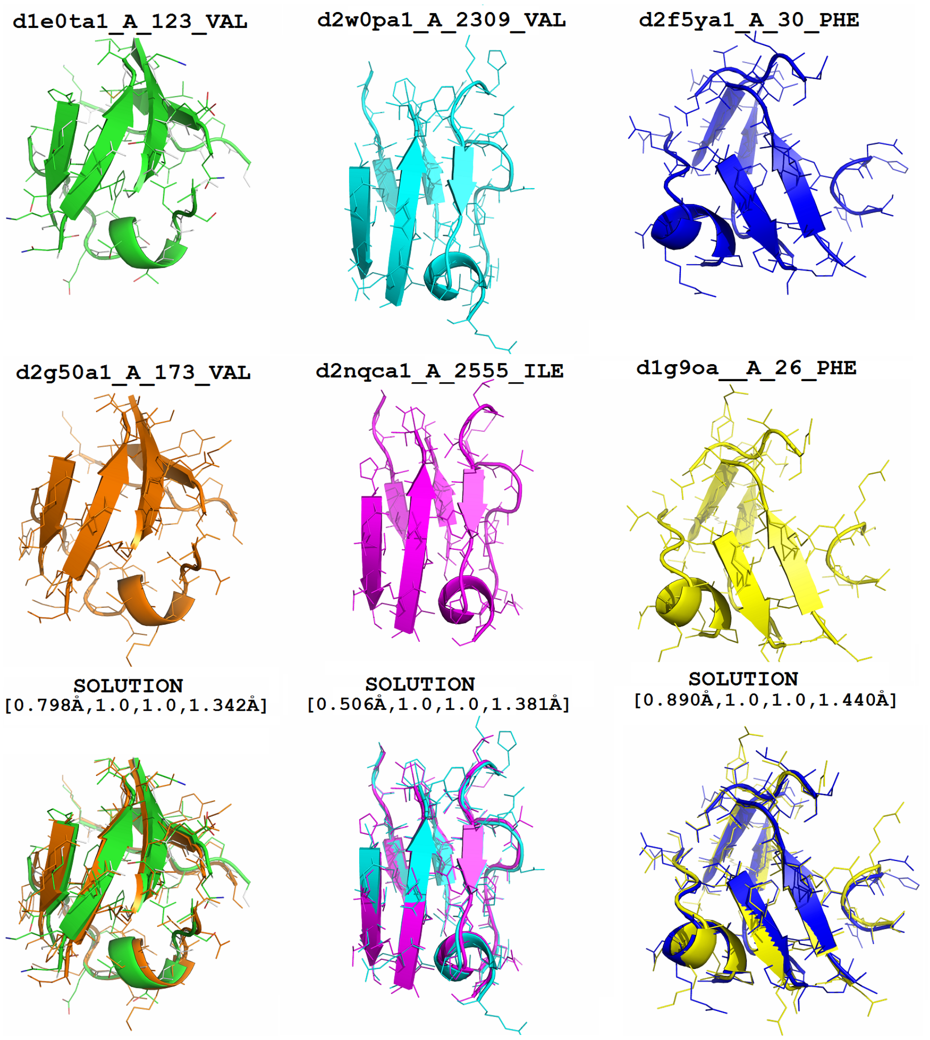

Figure Sf3: Visualization of instances of the problem and the corresponding optimal solutions. First two rows present descriptors that are compared and the third one is for their optimal structural alignment

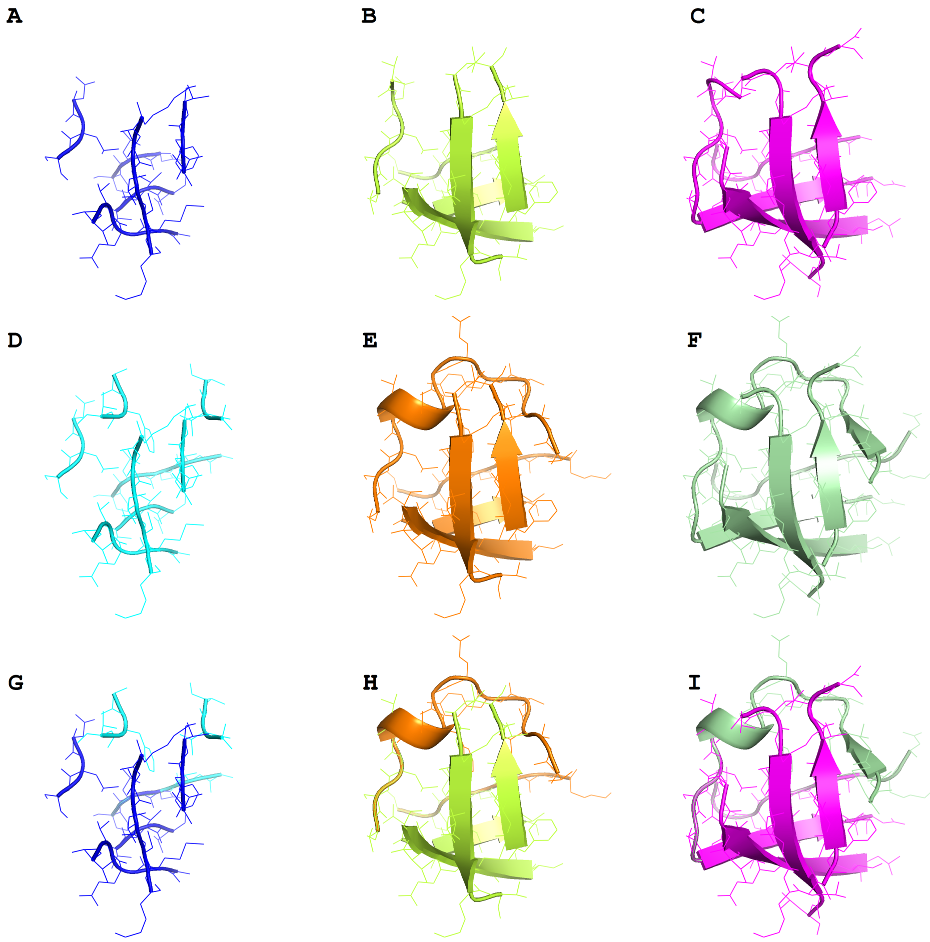

Figure Sf4: Visualization of structural motifs constructed in the proximity of residue A123-VAL (d1e0ta1) according to the building concept of descriptors taking into consideration the following values of element size: 3 [ES1], 5 [ES2], 7 [ES3], as well as two general types of expressions for identification of in-contact residues, namely "OR(DISTANCE:CBX $\leq$ 6.5, AND(DISTANCE:CBX $\leq$ DISTANCE:CA $-$ 0.75, DISTANCE:CBX $\leq$ 8.0))" [EXP1] and "OR(DISTANCE:SCGC $\leq$ 6.5, AND(DISTANCE:SCGC $\leq$ DISTANCE:CA $-$ 0.75, DISTANCE:SCGC $\leq$ 8.0))" [EXP2]. In these expressions, the following residue representatives are used: CA (C_α), CBX (C_β-extended point) (Hvidsten, Kryshtafovych and Fidelis, 2009), and SCGC (geometrical center of side-chain). Table St6 contains detailed information about the structural motifs. (A) Visualization prepared for parameters ES1, EXP1, (B) ES2, EXP1, (C) ES3, EXP1, (D) ES1, EXP2, (E) ES2, EXP2, (F) ES3, EXP2. (G) Visualization of superimposed 3D structures of motifs from figures A and D, (H) B and E, (I) C and F

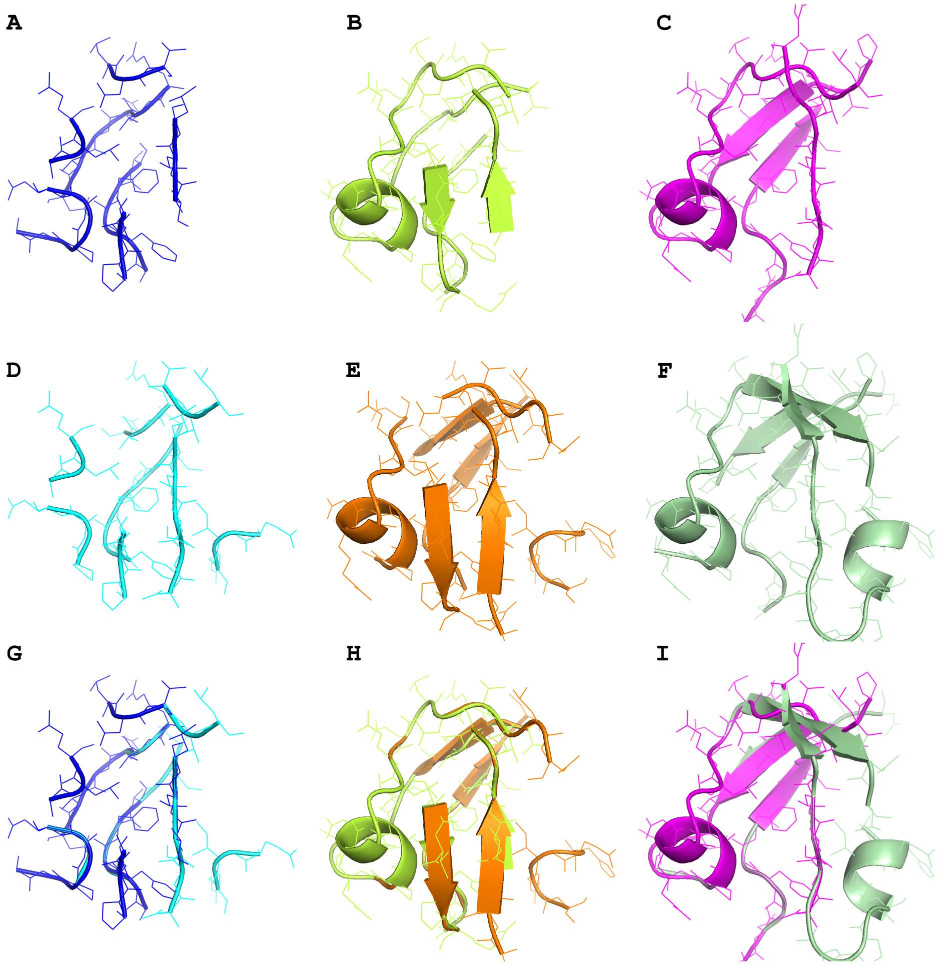

Figure Sf5: Visualization of structural motifs constructed in the proximity of residue A30-PHE (d2f5ya1) according to the building concept of descriptors taking into consideration the following values of element size: 3 [ES1], 5 [ES2], 7 [ES3], as well as two general types of expressions for identification of in-contact residues, namely "OR(DISTANCE:CBX $\leq$ 6.5, AND(DISTANCE:CBX $\leq$ DISTANCE:CA $-$ 0.75, DISTANCE:CBX $\leq$ 8.0))" [EXP1] and "OR(DISTANCE:SCGC $\leq$ 6.5, AND(DISTANCE:SCGC $\leq$ DISTANCE:CA $-$ 0.75, DISTANCE:SCGC $\leq$ 8.0))" [EXP2]. In these expressions, the following residue representatives are used: CA (C_α), CBX (C_β-extended point) (Hvidsten, Kryshtafovych and Fidelis, 2009), and SCGC (geometrical center of side-chain). Table St7 contains detailed information about the structural motifs. (A) Visualization prepared for parameters ES1, EXP1, (B) ES2, EXP1, (C) ES3, EXP1, (D) ES1, EXP2, (E) ES2, EXP2, (F) ES3, EXP2. (G) Visualization of superimposed 3D structures of motifs from figures A and D, (H) B and E, (I) C and F

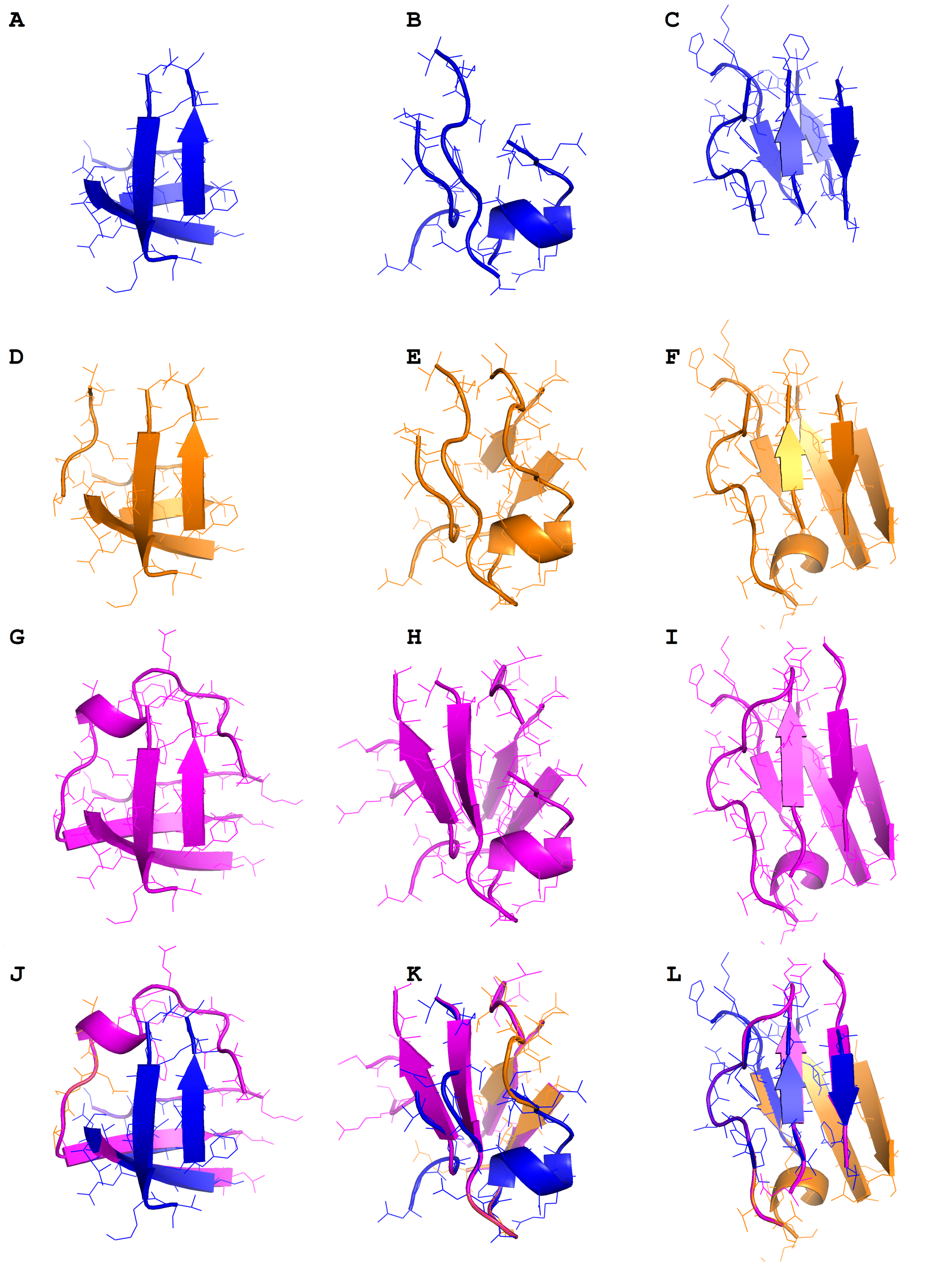

Figure Sf6: Visualization of 3D structures of descriptors constructed in the proximity of three example residues, namely A123-VAL (d1e0ta1) [RES1], A30-PHE (d2f5ya1) [RES2], and A2309-VAL (d2w0pa1) [RES3] by various expressions for identification of in-contact residues published in the literature. They are "DISTANCE:CBX $<$ 6.5" (Hvidsten *et al.*, 2003; Hvidsten, Laegreid *et al.*, 2009) [EXP1, in blue], "OR(DISTANCE:CBX $<$ 6.5, AND(DISTANCE:CBX $<$ DISTANCE:CA $-$ 0.75, DISTANCE:CBX $<$ 8.0))" (Hvidsten, Kryshtafovych and Fidelis, 2009) [EXP2, in orange], "OR(DISTANCE:CA $\leq$ 6.5, AND(DISTANCE:SCGC $\leq$ DISTANCE:CA $-$ 0.75, DISTANCE:SCGC $\leq$ 8.0))" (Daniluk and Lesyng, 2011) [EXP3, in magenta]. In these expressions, the following residue representatives are used: CA (C_α), CBX (C_β-extended point), and SCGC (geometrical center of side-chain). Table St8 contains detailed information about the descriptors. (A) Visualization prepared for parameters RES1, EXP1, (B) RES2, EXP1, (C) RES3, EXP1, (D) RES1, EXP2, (E) RES2, EXP2, (F) RES3, EXP2, (G) RES1, EXP3, (H) RES2, EXP3, (I) RES3, EXP3. (J–L) Visualization of superimposed 3D structures presented above

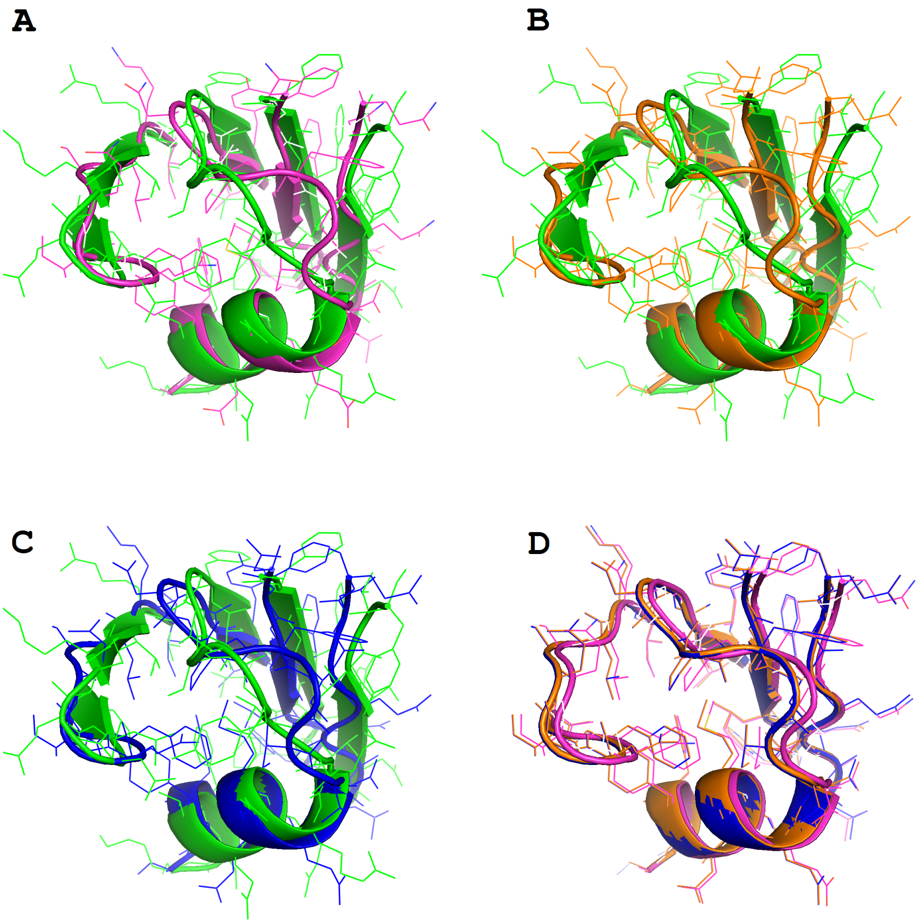

Figure Sf7: Visualization of structural alignments computed for descriptor pair d2cqca1_A_134_LEU, d1u2fa__A_18_MET. It is one of the most computationally interesting pairs listed in Table St5. The descriptors are composed of 11 elements. The tertiary structure of descriptor d2cqca1_A_134_LEU is depicted in green, and the corresponding structural alignment of descriptor d1u2fa__A_18_MET obtained because of the application of considered algorithms is depicted in magenta, orange, and blue, respectively. Table St9 contains detailed information about the descriptors and the resultant alignments. (A) Result of Algorithm 2 executed with the threshold parameter $f = 1.75$. (B) Result of Algorithm 3 executed with $f = 1.75$. (C) Result of Algorithm 4. (D) Visualization of all superimposed structural alignments of descriptor d1u2fa__A_18_MET

2 Detailed results

Table St1: Summary of solutions quality for Algorithm 1

	Algorithm 1 (1.75)					
descriptor elements count	coverage of similar descriptor pairs [%]	quality identity [%]	higher global RMSD, equal residues ratio [%]	lower residues ratio [%]	global RMSD [Å]	
					avg.	std. dev.
3	36.47	100.00	0.00	0.00	1.50	0.33
4	52.00	100.00	0.00	0.00	1.76	0.45
5	86.97	92.75	1.93	5.31	2.02	0.58
6	83.33	92.50	2.50	5.00	1.96	0.58
7	85.32	100.00	0.00	0.00	2.03	0.56
8	82.05	96.88	1.04	2.08	2.15	0.49
9	80.79	96.95	1.22	1.83	2.03	0.57
10	61.71	94.44	3.24	2.31	2.39	0.60
11	66.75	94.31	2.85	2.85	2.26	0.52

	Algorithm 1 (2.0)					
descriptor elements count	coverage of similar descriptor pairs [%]	quality identity [%]	higher global RMSD, equal residues ratio [%]	lower residues ratio [%]	global RMSD [Å]	
					avg.	std. dev.
3	47.35	100.00	0.00	0.00	1.65	0.41
4	72.00	100.00	0.00	0.00	1.98	0.55
5	93.70	95.96	1.79	2.24	2.15	0.65
6	90.28	96.15	2.31	1.54	2.10	0.67
7	88.99	97.94	1.03	1.03	2.09	0.59
8	88.03	97.09	0.97	1.94	2.25	0.56
9	86.70	98.86	1.14	0.00	2.13	0.63
10	62.86	96.82	3.18	0.00	2.42	0.62
11	67.93	96.15	2.45	1.40	2.29	0.54

	Algorithm 1 (2.33)					
descriptor elements count	coverage of similar descriptor pairs [%]	quality identity [%]	higher global RMSD, equal residues ratio [%]	lower residues ratio [%]	global RMSD [Å]	
					avg.	std. dev.
3	70.88	100.00	0.00	0.00	1.99	0.60
4	92.00	100.00	0.00	0.00	2.20	0.65
5	91.60	96.79	1.83	1.38	2.15	0.66
6	90.97	97.71	2.29	0.00	2.12	0.69
7	88.99	100.00	0.00	0.00	2.10	0.61
8	85.47	99.00	1.00	0.00	2.24	0.56
9	87.19	98.87	1.13	0.00	2.14	0.64
10	62.29	96.79	3.21	0.00	2.42	0.62
11	67.70	96.84	2.46	0.70	2.28	0.54

Table St2: Summary of solutions quality for Algorithm 2

	**Algorithm 2 (1.75)**					
descriptor elements count	coverage of similar descriptor pairs [%]	quality identity [%]	higher global RMSD, equal residues ratio [%]	lower residues ratio [%]	global RMSD [Å] avg.	global RMSD [Å] std. dev.
3	36.47	100.00	0.00	0.00	1.50	0.33
4	52.00	100.00	0.00	0.00	1.76	0.45
5	88.24	92.38	1.90	5.71	2.02	0.58
6	84.72	92.62	2.46	4.92	1.95	0.58
7	88.99	100.00	0.00	0.00	2.02	0.55
8	86.32	95.05	0.99	3.96	2.12	0.50
9	85.22	95.38	2.31	2.31	2.04	0.57
10	75.43	92.05	4.17	3.79	2.35	0.58
11	79.33	91.62	3.89	4.49	2.26	0.51

	**Algorithm 2 (2.0)**					
descriptor elements count	coverage of similar descriptor pairs [%]	quality identity [%]	higher global RMSD, equal residues ratio [%]	lower residues ratio [%]	global RMSD [Å] avg.	global RMSD [Å] std. dev.
3	47.35	100.00	0.00	0.00	1.65	0.41
4	72.00	100.00	0.00	0.00	1.98	0.55
5	96.64	95.65	1.74	2.61	2.13	0.64
6	93.75	96.30	2.22	1.48	2.09	0.66
7	96.33	98.10	0.95	0.95	2.09	0.59
8	97.44	95.61	0.88	3.51	2.23	0.56
9	94.09	96.34	2.62	1.05	2.14	0.63
10	86.00	90.37	4.98	4.65	2.45	0.60
11	86.70	91.78	3.56	4.66	2.32	0.54

	**Algorithm 2 (2.33)**					
descriptor elements count	coverage of similar descriptor pairs [%]	quality identity [%]	higher global RMSD, equal residues ratio [%]	lower residues ratio [%]	global RMSD [Å] avg.	global RMSD [Å] std. dev.
3	70.88	100.00	0.00	0.00	1.99	0.60
4	92.00	100.00	0.00	0.00	2.20	0.65
5	96.64	96.52	1.74	1.74	2.14	0.65
6	95.14	97.81	2.19	0.00	2.11	0.68
7	97.25	99.06	0.94	0.00	2.11	0.60
8	97.44	97.37	0.88	1.75	2.23	0.56
9	95.07	96.37	2.59	1.04	2.15	0.63
10	88.86	89.39	5.47	5.14	2.48	0.61
11	88.84	91.44	3.74	4.81	2.35	0.55

Table St3: Summary of solutions quality for Algorithm 3

	**Algorithm 3 (1.75)**					
descriptor elements count	coverage of similar descriptor pairs [%]	quality identity [%]	higher global RMSD, equal residues ratio [%]	lower residues ratio [%]	global RMSD [Å] avg.	global RMSD [Å] std. dev.
3	36.47	100.00	0.00	0.00	1.50	0.33
4	52.00	100.00	0.00	0.00	1.76	0.45
5	89.50	91.55	2.35	6.10	2.02	0.58
6	88.89	87.50	4.69	7.81	2.01	0.61
7	90.83	97.98	1.01	1.01	2.03	0.56
8	92.31	90.74	2.78	6.48	2.17	0.53
9	89.16	92.82	3.87	3.31	2.08	0.58
10	91.43	83.13	10.00	6.88	2.51	0.62
11	93.11	85.20	5.10	9.69	2.36	0.55

	**Algorithm 3 (2.0)**					
descriptor elements count	coverage of similar descriptor pairs [%]	quality identity [%]	higher global RMSD, equal residues ratio [%]	lower residues ratio [%]	global RMSD [Å] avg.	global RMSD [Å] std. dev.
3	47.35	100.00	0.00	0.00	1.65	0.41
4	72.00	100.00	0.00	0.00	1.98	0.55
5	97.06	95.24	2.16	2.60	2.14	0.65
6	96.53	92.81	4.32	2.88	2.13	0.68
7	96.33	98.10	0.95	0.95	2.09	0.59
8	97.44	93.86	2.63	3.51	2.23	0.57
9	97.04	94.92	3.55	1.52	2.17	0.63
10	92.29	85.76	9.29	4.95	2.52	0.62
11	95.25	88.53	4.99	6.48	2.38	0.56

	**Algorithm 3 (2.33)**					
descriptor elements count	coverage of similar descriptor pairs [%]	quality identity [%]	higher global RMSD, equal residues ratio [%]	lower residues ratio [%]	global RMSD [Å] avg.	global RMSD [Å] std. dev.
3	70.88	100.00	0.00	0.00	1.99	0.60
4	92.00	100.00	0.00	0.00	2.20	0.65
5	97.06	96.10	2.16	1.73	2.15	0.66
6	97.22	95.00	4.29	0.71	2.15	0.70
7	97.25	99.06	0.94	0.00	2.11	0.61
8	97.44	94.74	2.63	2.63	2.23	0.57
9	98.03	94.97	3.52	1.51	2.18	0.64
10	92.29	86.07	9.29	4.64	2.52	0.62
11	95.49	89.05	4.73	6.22	2.38	0.56

Table St4: Top 10 computationally expensive descriptor pairs (of longest processing times of structural comparison done by Algorithm 4). The descriptors were generated with the following expression for identification of in-contact residues: OR(DISTANCE:SCGC $\leq$ 6.5, AND(DISTANCE:SCGC $\leq$ DISTANCE:CA $-$ 0.75, DISTANCE:SCGC $\leq$ 8.0)). The structural comparisons were performed with CA (C_α) and SCGC (geometrical center of side-chain) as representative atom names. All descriptors are composed of 11 elements

descriptor pair	algorithm (threshold)	aligned elements [%]	aligned residues [%]	global RMSD [Å]	processing time [ms]
d1x5oa1_A_46_LEU d2cpza1_A_421_PHE	1 (1.75)	100.00	100.00	1.92	7
	1 (2.0)	100.00	100.00	1.92	8
	1 (2.33)	100.00	100.00	1.92	7
	2 (1.75)	100.00	100.00	1.92	39
	2 (2.0)	100.00	100.00	1.92	39
	2 (2.33)	100.00	100.00	1.92	40
	3 (1.75)	100.00	100.00	1.92	146
	3 (2.0)	100.00	100.00	1.92	147
	3 (2.33)	100.00	100.00	1.92	148
	4	100.00	100.00	1.92	4526764

descriptor pair	algorithm (threshold)	aligned elements [%]	aligned residues [%]	global RMSD [Å]	processing time [ms]
d1qg3a1_A_1183_LEU d2cuha1_A_63_LEU	1 (1.75)	–	–	–	7
	1 (2.0)	–	–	–	6
	1 (2.33)	–	–	–	6
	2 (1.75)	81.82	82.93	2.14	29
	2 (2.0)	81.82	82.93	2.14	47
	2 (2.33)	81.82	82.93	2.14	48
	3 (1.75)	81.82	82.93	2.14	144
	3 (2.0)	81.82	82.93	2.14	149
	3 (2.33)	81.82	82.93	2.14	149
	4	90.91	95.12	3.29	2337319

descriptor pair	algorithm (threshold)	aligned elements [%]	aligned residues [%]	global RMSD [Å]	processing time [ms]
d1x4aa1_A_39_ILE d2cpea1_A_377_LEU	1 (1.75)	100.00	100.00	2.01	7
	1 (2.0)	100.00	100.00	2.01	7
	1 (2.33)	100.00	100.00	2.01	7
	2 (1.75)	100.00	100.00	2.01	41
	2 (2.0)	100.00	100.00	2.01	41
	2 (2.33)	100.00	100.00	2.01	42
	3 (1.75)	100.00	100.00	2.01	150
	3 (2.0)	100.00	100.00	2.01	151
	3 (2.33)	100.00	100.00	2.01	150
	4	100.00	100.00	2.01	2227151

descriptor pair	algorithm (threshold)	aligned elements [%]	aligned residues [%]	global RMSD [Å]	processing time [ms]
d1g9oa__A_59_LEU d1wf8a1_A_65_ILE	1 (1.75)	100.00	100.00	1.91	7
	1 (2.0)	100.00	100.00	1.91	7
	1 (2.33)	100.00	100.00	1.91	7
	2 (1.75)	100.00	100.00	1.91	38
	2 (2.0)	100.00	100.00	1.91	39
	2 (2.33)	100.00	100.00	1.91	39
	3 (1.75)	100.00	100.00	1.91	150
	3 (2.0)	100.00	100.00	1.91	149
	3 (2.33)	100.00	100.00	1.91	152
	4	100.00	100.00	1.91	2121192

descriptor pair	algorithm (threshold)	aligned elements [%]	aligned residues [%]	global RMSD [Å]	processing time [ms]
d2cdqa2_A_344_LEU d2hmfa3_A_321_ILE	1 (1.75)	–	–	–	6
	1 (2.0)	–	–	–	7
	1 (2.33)	–	–	–	7
	2 (1.75)	81.82	79.07	3.15	26
	2 (2.0)	81.82	79.07	3.15	44
	2 (2.33)	81.82	79.07	3.15	44
	3 (1.75)	81.82	79.07	3.15	144
	3 (2.0)	81.82	79.07	3.15	151
	3 (2.33)	81.82	79.07	3.15	150
	4	81.82	83.72	3.01	2113848

descriptor pair	algorithm (threshold)	aligned elements [%]	aligned residues [%]	global RMSD [Å]	processing time [ms]
d1fnha2_A_150_ILE d2fnba__A_64_VAL	1 (1.75)	100.00	100.00	2.88	6
	1 (2.0)	100.00	100.00	2.88	6
	1 (2.33)	100.00	100.00	2.88	6
	2 (1.75)	100.00	100.00	2.88	39
	2 (2.0)	100.00	100.00	2.88	39
	2 (2.33)	100.00	100.00	2.88	39
	3 (1.75)	100.00	100.00	2.88	168
	3 (2.0)	100.00	100.00	2.88	168
	3 (2.33)	100.00	100.00	2.88	168
	4	100.00	100.00	2.88	2005195

descriptor pair	algorithm (threshold)	aligned elements [%]	aligned residues [%]	global RMSD [Å]	processing time [ms]
d1cwva1_A_524_VAL d2h26a1_A_206_CYS	1 (1.75)	–	–	–	9
	1 (2.0)	–	–	–	10
	1 (2.33)	–	–	–	9
	2 (1.75)	81.82	90.00	2.05	29
	2 (2.0)	81.82	90.00	2.05	47
	2 (2.33)	81.82	90.00	2.05	46
	3 (1.75)	90.91	95.00	3.19	149
	3 (2.0)	90.91	95.00	3.19	153
	3 (2.33)	90.91	95.00	3.19	159
	4	90.91	95.00	2.99	1951039

descriptor pair	algorithm (threshold)	aligned elements [%]	aligned residues [%]	global RMSD [Å]	processing time [ms]
d1fc6a3_A_199_ILE d1rzxa__A_218_VAL	1 (1.75)	100.00	100.00	1.70	6
	1 (2.0)	100.00	100.00	1.70	7
	1 (2.33)	100.00	100.00	1.70	6
	2 (1.75)	100.00	100.00	1.70	38
	2 (2.0)	100.00	100.00	1.70	39
	2 (2.33)	100.00	100.00	1.70	39
	3 (1.75)	100.00	100.00	1.70	150
	3 (2.0)	100.00	100.00	1.70	151
	3 (2.33)	100.00	100.00	1.70	150
	4	100.00	100.00	1.70	1896075

descriptor pair	algorithm (threshold)	aligned elements [%]	aligned residues [%]	global RMSD [Å]	processing time [ms]
d2cqca1_A_134_LEU d1x4aa1_A_39_ILE	1 (1.75)	100.00	100.00	1.86	10
	1 (2.0)	100.00	100.00	1.86	10
	1 (2.33)	100.00	100.00	1.86	10
	2 (1.75)	100.00	100.00	1.86	42
	2 (2.0)	100.00	100.00	1.86	41
	2 (2.33)	100.00	100.00	1.86	42
	3 (1.75)	100.00	100.00	1.86	152
	3 (2.0)	100.00	100.00	1.86	152
	3 (2.33)	100.00	100.00	1.86	152
	4	100.00	100.00	1.86	1616547

descriptor pair	algorithm (threshold)	aligned elements [%]	aligned residues [%]	global RMSD [Å]	processing time [ms]
d1cvja1_A_27_LEU d2cqca1_A_134_LEU	1 (1.75)	100.00	100.00	1.91	7
	1 (2.0)	100.00	100.00	1.91	7
	1 (2.33)	100.00	100.00	1.91	7
	2 (1.75)	100.00	100.00	1.91	38
	2 (2.0)	100.00	100.00	1.91	38
	2 (2.33)	100.00	100.00	1.91	38
	3 (1.75)	100.00	100.00	1.91	146
	3 (2.0)	100.00	100.00	1.91	147
	3 (2.33)	100.00	100.00	1.91	149
	4	100.00	100.00	1.91	1424952

Table St5: Some of the most computationally interesting descriptor pairs, i.e., the ones of the greatest variability within the algorithms' output (in the sense of aligned residues). Each descriptor size (number of its elements) is represented by one pair. The descriptors were generated with the following expression for identification of in-contact residues: OR(DISTANCE:SCGC $\leq$ 6.5, AND(DISTANCE:SCGC $\leq$ DISTANCE:CA $-$ 0.75, DISTANCE:SCGC $\leq$ 8.0)). The structural comparisons were performed with CA (C_α) and SCGC (geometrical center of side-chain) as representative atom names

descriptor pair (size)	algorithm (threshold)	aligned elements [%]	aligned residues [%]	global RMSD [Å]	processing time [ms]
d3b5ha1_A_199_THR d1lp9e1_E_111_SER (3)	1 (1.75)	–	–	–	1
	1 (2.0)	100.00	100.00	2.23	4
	1 (2.33)	100.00	100.00	2.23	5
	2 (1.75)	–	–	–	1
	2 (2.0)	100.00	100.00	2.23	8
	2 (2.33)	100.00	100.00	2.23	8
	3 (1.75)	–	–	–	4
	3 (2.0)	100.00	100.00	2.23	10
	3 (2.33)	100.00	100.00	2.23	10
	4	100.00	100.00	2.23	6

descriptor pair (size)	algorithm (threshold)	aligned elements [%]	aligned residues [%]	global RMSD [Å]	processing time [ms]
d3deoa1_A_93_ARG d1tvxa__A_43_GLU (4)	1 (1.75)	–	–	–	1
	1 (2.0)	100.00	100.00	2.54	5
	1 (2.33)	100.00	100.00	2.54	5
	2 (1.75)	–	–	–	1
	2 (2.0)	100.00	100.00	2.54	9
	2 (2.33)	100.00	100.00	2.54	9
	3 (1.75)	–	–	–	7
	3 (2.0)	100.00	100.00	2.54	15
	3 (2.33)	100.00	100.00	2.54	14
	4	100.00	100.00	2.54	7

descriptor pair (size)	algorithm (threshold)	aligned elements [%]	aligned residues [%]	global RMSD [Å]	processing time [ms]
d1n4na__A_4_LYS d2j0wa3_A_388_LEU (5)	1 (1.75)	–	–	–	1
	1 (2.0)	80.00	73.68	2.50	5
	1 (2.33)	100.00	100.00	3.38	5
	2 (1.75)	–	–	–	1
	2 (2.0)	80.00	73.68	2.50	4
	2 (2.33)	100.00	100.00	3.38	9
	3 (1.75)	80.00	73.68	2.50	14
	3 (2.0)	80.00	73.68	2.50	18
	3 (2.33)	100.00	100.00	3.38	27
	4	100.00	100.00	3.38	29

descriptor pair (size)	algorithm (threshold)	aligned elements [%]	aligned residues [%]	global RMSD [Å]	processing time [ms]
d2hjqa2_A_3_THR d1g2913_1_250_ALA (6)	1 (1.75)	–	–	–	1
	1 (2.0)	83.33	90.00	2.76	6
	1 (2.33)	100.00	100.00	3.06	7
	2 (1.75)	–	–	–	1
	2 (2.0)	83.33	90.00	2.76	10
	2 (2.33)	100.00	100.00	3.06	16
	3 (1.75)	83.33	90.00	2.76	27
	3 (2.0)	83.33	90.00	2.76	38
	3 (2.33)	100.00	100.00	3.06	50
	4	100.00	100.00	3.06	57

descriptor pair (size)	algorithm (threshold)	aligned elements [%]	aligned residues [%]	global RMSD [Å]	processing time [ms]
d2fkla1_A_153_VAL d2fkla1_A_157_THR (7)	1 (1.75)	–	–	–	1
	1 (2.0)	85.71	85.19	2.55	6
	1 (2.33)	100.00	100.00	2.93	8
	2 (1.75)	–	–	–	2
	2 (2.0)	85.71	85.19	2.55	12
	2 (2.33)	100.00	100.00	2.93	18
	3 (1.75)	85.71	85.19	2.55	46
	3 (2.0)	85.71	85.19	2.55	59
	3 (2.33)	100.00	100.00	2.93	73
	4	100.00	100.00	2.93	676

descriptor pair (size)	algorithm (threshold)	aligned elements [%]	aligned residues [%]	global RMSD [Å]	processing time [ms]
d1bnba__A_10_GLY d1fd3a__A_13_ALA (8)	1 (1.75)	–	–	–	1
	1 (2.0)	87.50	89.66	2.96	6
	1 (2.33)	100.00	100.00	3.15	6
	2 (1.75)	–	–	–	1
	2 (2.0)	87.50	89.66	2.96	10
	2 (2.33)	100.00	100.00	3.15	18
	3 (1.75)	87.50	89.66	2.96	70
	3 (2.0)	87.50	89.66	2.96	89
	3 (2.33)	100.00	100.00	3.15	102
	4	100.00	100.00	3.15	18

descriptor pair (size)	algorithm (threshold)	aligned elements [%]	aligned residues [%]	global RMSD [Å]	processing time [ms]
d2d9ta1_A_43_VAL d2pu9b1_B_55_VAL (9)	1 (1.75)	88.89	93.94	2.65	6
	1 (2.0)	100.00	100.00	3.49	7
	1 (2.33)	100.00	100.00	3.49	6
	2 (1.75)	88.89	93.94	2.65	11
	2 (2.0)	100.00	100.00	3.49	20
	2 (2.33)	100.00	100.00	3.49	20
	3 (1.75)	88.89	93.94	2.65	103
	3 (2.0)	100.00	100.00	3.49	114
	3 (2.33)	100.00	100.00	3.49	114
	4	100.00	100.00	3.49	3198

descriptor pair (size)	algorithm (threshold)	aligned elements [%]	aligned residues [%]	global RMSD [Å]	processing time [ms]
d2c60a1_A_49_PHE d1k8rb__B_75_PHE (10)	1 (1.75)	90.00	94.87	2.57	7
	1 (2.0)	100.00	100.00	2.73	6
	1 (2.33)	100.00	100.00	2.73	8
	2 (1.75)	90.00	94.87	2.57	14
	2 (2.0)	100.00	100.00	2.73	24
	2 (2.33)	100.00	100.00	2.73	23
	3 (1.75)	90.00	94.87	2.57	133
	3 (2.0)	100.00	100.00	2.73	139
	3 (2.33)	100.00	100.00	2.73	141
	4	100.00	100.00	2.73	48766

descriptor pair (size)	algorithm (threshold)	aligned elements [%]	aligned residues [%]	global RMSD [Å]	processing time [ms]
d2cqca1_A_134_LEU d1u2fa__A_18_MET (11)	1 (1.75)	81.82	84.44	2.09	7
	1 (2.0)	100.00	100.00	2.55	7
	1 (2.33)	100.00	100.00	2.55	7
	2 (1.75)	81.82	84.44	2.09	14
	2 (2.0)	100.00	100.00	2.55	39
	2 (2.33)	100.00	100.00	2.55	40
	3 (1.75)	90.91	95.56	2.59	142
	3 (2.0)	100.00	100.00	2.55	155
	3 (2.33)	100.00	100.00	2.55	150
	4	100.00	100.00	2.55	352344

Table St6: Details of structural motifs constructed in the proximity of residue A123-VAL (d1e0ta1) according to the building concept of descriptors taking into consideration values 3, 5, 7 of element size and two types of expressions for identification of in-contact residues: "OR(DISTANCE:CBX $\leq$ 6.5, AND(DISTANCE:CBX $\leq$ DISTANCE:CA $-$ 0.75, DISTANCE:CBX $\leq$ 8.0))" [EXP1] and "OR(DISTANCE:SCGC $\leq$ 6.5, AND(DISTANCE:SCGC $\leq$ DISTANCE:CA $-$ 0.75, DISTANCE:SCGC $\leq$ 8.0))" [EXP2]. See Figure Sf4 for visualization of 3D structures of these motifs

expression	element size	segments count	elements count	residues count	position	sequence
EXP1	3	6	8	22	A92–A94	TFT
					A116–A118	DLS
					A122–A124	TVL
					A131–A135	GMEVT
					A141–A145	KVICK
					A159–A161	NLP
	5	6	8	34	A91–A95	FTFTT
					A115–A119	TDLSV
					A121–A125	NTVLV
					A130–A136	IGMEVTA
					A140–A146	NKVICKV
					A158–A162	VNLPG
	7	5	8	45	A90–A96	TFTFTTD
					A114–A126	TTDLSVGNTVLVD
					A129–A137	LIGMEVTAI
					A139–A147	GNKVICKVL
					A157–A163	GVNLPGV
EXP2	3	8	11	30	A92–A94	TFT
					A107–A109	AVT
					A112–A114	GFT
					A116–A118	DLS
					A122–A124	TVL
					A131–A135	GMEVT
					A141–A145	KVICK
					A157–A161	GVNLP
	5	6	11	45	A91–A95	FTFTT
					A106–A119	VAVTYEGFTTDLSV
					A121–A125	NTVLV
					A130–A136	IGMEVTA
					A140–A146	NKVICKV
					A156–A162	KGVNLPG
	7	5	11	56	A90–A96	TFTFTTD
					A105–A126	MVAVTYEGFTTDLSVGNTVLVD
					A129–A137	LIGMEVTAI
					A139–A147	GNKVICKVL
					A155–A163	NKGVNLPGV

Table St7: Details of structural motifs constructed in the proximity of residue A30-PHE (d2f5ya1) according to the building concept of descriptors taking into consideration values 3, 5, 7 of element size and two types of expressions for identification of in-contact residues: "OR(DISTANCE:CBX $\leq$ 6.5, AND(DISTANCE:CBX $\leq$ DISTANCE:CA − 0.75, DISTANCE:CBX $\leq$ 8.0))" [EXP1] and "OR(DISTANCE:SCGC $\leq$ 6.5, AND(DISTANCE:SCGC $\leq$ DISTANCE:CA − 0.75, DISTANCE:SCGC $\leq$ 8.0))" [EXP2]. See Figure Sf5 for visualization of 3D structures of these motifs

expression	element size	segments count	elements count	residues count	position	sequence
EXP1	3	7	10	28	A20–A22<br>A27–A31<br>A39–A44<br>A46–A50<br>A53–A55<br>A57–A59<br>A88–A90	TIP<br>GFGFT<br>RVQAVD<br>GGPAE<br>GLQ<br>LDT<br>ILL
	5	4	10	40	A19–A23<br>A26–A32<br>A38–A60<br>A87–A91	ITIPR<br>DGFGFTI<br>VRVQAVDSGGPAERAGLQQLDTV<br>IILLV
	7	3	7	41	A25–A33<br>A37–A61<br>A86–A92	KDGFGFTIC<br>PVRVQAVDSGGPAERAGLQQLDTVL<br>EIILLVW
EXP2	3	8	11	29	A20–A22<br>A28–A33<br>A39–A41<br>A48–A50<br>A53–A55<br>A59–A61<br>A79–A81<br>A86–A90	TIP<br>FGFTIC<br>RVQ<br>PAE<br>GLQ<br>TVL<br>EIR<br>EIILL
	5	7	11	45	A19–A23<br>A27–A34<br>A38–A42<br>A47–A56<br>A58–A62<br>A78–A82<br>A85–A91	ITIPR<br>GFGFTICC<br>VRVQA<br>GPAERAGLQQ<br>DTVLQ<br>HEIRS<br>SEIILLV
	7	4	10	51	A26–A35<br>A37–A43<br>A46–A63<br>A77–A92	DGFGFTICCD<br>PVRVQAV<br>GGPAERAGLQQLDTVLQL<br>AHEIRSCPSEIILLVW

Table St8: Details of descriptors constructed in the proximity of residues A123-VAL (d1e0ta1), A30-PHE (d2f5ya1), and A2309-VAL (d2w0pa1) by various expressions for identification of in-contact residues published in the literature. See Figure Sf6 for the definitions of the expressions and for visualization of 3D structures of these descriptors

descriptor	expression	segments count	elements count	residues count	position	sequence
d1e0ta1_A_123_VAL	EXP1	5	7	29	A91–A95<br>A121–A125<br>A130–A136<br>A140–A146<br>A158–A162	FTFTT<br>NTVLV<br>IGMEVTA<br>NKVICKV<br>VNLPG
	EXP2	6	8	34	A91–A95<br>A115–A119<br>A121–A125<br>A130–A136<br>A140–A146<br>A158–A162	FTFTT<br>TDLSV<br>NTVLV<br>IGMEVTA<br>NKVICKV<br>VNLPG
	EXP3	5	13	48	A91–A95<br>A106–A126<br>A129–A136<br>A140–A146<br>A156–A162	FTFTT<br>VAVTYEGFTTDLSVGNTVLVD<br>LIGMEVTA<br>NKVICKV<br>KGVNLPG
d2f5ya1_A_30_PHE	EXP1	3	6	25	A26–A32<br>A38–A45<br>A47–A56	DGFGFTI<br>VRVQAVDS<br>GPAERAGLQQ
	EXP2	4	10	40	A19–A23<br>A26–A32<br>A38–A60<br>A87–A91	ITIPR<br>DGFGFTI<br>VRVQAVDSGGPAERAGLQQLDTV<br>IILLV
	EXP3	6	12	50	A19–A23<br>A26–A34<br>A38–A56<br>A58–A62<br>A78–A82<br>A85–A91	ITIPR<br>DGFGFTICC<br>VRVQAVDSGGPAERAGLQQ<br>DTVLQ<br>HEIRS<br>SEIILLV
d2w0pa1_A_2309_VAL	EXP1	5	7	30	A2239–A2245<br>A2259–A2263<br>A2271–A2275<br>A2307–A2311<br>A2314–A2321	HKVRAGG<br>FSIWT<br>LAIAV<br>VSVKF<br>EHIPDSPF
	EXP2	6	10	44	A2239–A2250<br>A2257–A2263<br>A2271–A2275<br>A2293–A2297<br>A2307–A2311<br>A2314–A2323	HKVRAGGPGLER<br>AEFSIWT<br>LAIAV<br>CGVAY<br>VSVKF<br>EHIPDSPFVV
	EXP3	5	14	49	A2239–A2250<br>A2257–A2263<br>A2269–A2275<br>A2293–A2297<br>A2306–A2323	HKVRAGGPGLER<br>AEFSIWT<br>GGLAIAV<br>CGVAY<br>EVSVKFNEEHIPDSPFVV

Table St9: Details of descriptors d2cqca1_A_134_LEU, d1u2fa__A_18_MET, and their structural alignments obtained as results of Algorithms 1–4. This pair of descriptors is the greatest one among the pairs presented in Table St5. See Figure Sf7 for visualization of these structural alignments

descriptor	segments count	elements count	residues count	position	sequence
d2cqca1_A_134_LEU	4	11	45	A120–A140<br>A145–A151<br>A160–A166<br>A182–A191	LGVFGLSLYTTERDLREVFSK<br>ADVSIVY<br>GFAFVYF<br>MELDGRRIRV
d1u2fa__A_18_MET	4	11	45	A4–A24<br>A40–A46<br>A49–A55<br>A70–A79	LYVGNIPFGITEEAMMDFFNA<br>LAVQINQ<br>NFAFLEF<br>IIFQGQSLKI

algorithm (threshold)	aligned elements [%]	aligned residues [%]	global RMSD [Å]	alignment
1 (1.75)	81.82	84.44	2.09	LGVFG.......LSLYTTERDLREVFSKADVSIVYGFAFVYFMELDGRRIRV<br>LYVGNIPFGITE.......EAMMDFFNALAVQINQNFAFLEFIIFQGQSLKI
1 (2.0)	100.00	100.00	2.55	LGVFGLSLYTTERDLREVFSKADVSIVYGFAFVYFMELDGRRIRV<br>LYVGNIPFGITEEAMMDFFNALAVQINQNFAFLEFIIFQGQSLKI
1 (2.33)	100.00	100.00	2.55	LGVFGLSLYTTERDLREVFSKADVSIVYGFAFVYFMELDGRRIRV<br>LYVGNIPFGITEEAMMDFFNALAVQINQNFAFLEFIIFQGQSLKI
2 (1.75)	81.82	84.44	2.09	LGVFG.......LSLYTTERDLREVFSKADVSIVYGFAFVYFMELDGRRIRV<br>LYVGNIPFGITE.......EAMMDFFNALAVQINQNFAFLEFIIFQGQSLKI
2 (2.0)	100.00	100.00	2.55	LGVFGLSLYTTERDLREVFSKADVSIVYGFAFVYFMELDGRRIRV<br>LYVGNIPFGITEEAMMDFFNALAVQINQNFAFLEFIIFQGQSLKI
2 (2.33)	100.00	100.00	2.55	LGVFGLSLYTTERDLREVFSKADVSIVYGFAFVYFMELDGRRIRV<br>LYVGNIPFGITEEAMMDFFNALAVQINQNFAFLEFIIFQGQSLKI
3 (1.75)	90.91	95.56	2.59	LGVFG..LSLYTTERDLREVFSKADVSIVYGFAFVYFMELDGRRIRV<br>LYVGNIP..FGITEEAMMDFFNALAVQINQNFAFLEFIIFQGQSLKI
3 (2.0)	100.00	100.00	2.55	LGVFGLSLYTTERDLREVFSKADVSIVYGFAFVYFMELDGRRIRV<br>LYVGNIPFGITEEAMMDFFNALAVQINQNFAFLEFIIFQGQSLKI
3 (2.33)	100.00	100.00	2.55	LGVFGLSLYTTERDLREVFSKADVSIVYGFAFVYFMELDGRRIRV<br>LYVGNIPFGITEEAMMDFFNALAVQINQNFAFLEFIIFQGQSLKI
4	100.00	100.00	2.55	LGVFGLSLYTTERDLREVFSKADVSIVYGFAFVYFMELDGRRIRV<br>LYVGNIPFGITEEAMMDFFNALAVQINQNFAFLEFIIFQGQSLKI
